# How ligand binds to the type 1 insulin-like growth factor receptor

**DOI:** 10.1038/s41467-018-03219-7

**Published:** 2018-02-26

**Authors:** Yibin Xu, Geoffrey K.-W. Kong, John G. Menting, Mai B. Margetts, Carlie A. Delaine, Lauren M. Jenkin, Vladislav V. Kiselyov, Pierre De Meyts, Briony E. Forbes, Michael C. Lawrence

**Affiliations:** 1 The Walter and Eliza Hall Institute of Medical Research, 1G Royal Parade, Parkville, VIC 3052 Australia; 2 Department of Medical Biology, University of Melbourne, Royal Parade, Parkville, VIC 3050 Australia; 3 Discipline of Medical Biochemistry, College of Medicine and Public Health, Flinders University of South Australia, Bedford Park, SA 5042 Australia; 4 Lilly Research Laboratories, Eli Lilly and Company, Indianapolis, IN 46285 USA; 5 Department of Cell Signaling, de Duve Institute, B-1200 Brussels, Belgium; 6 Department of Stem Cell Research, Novo Nordisk A/S, 2760 Måløv, Denmark; 7 De Meyts R&D Consulting, Avenue Reine Astrid 42, B-1950 Kraainem, Belgium; 8 Present Address: Monash Macromolecular Crystallisation Facility, 11 Chancellors Walk, Clayton Campus, Monash University, Clayton, VIC 3800 Australia

## Abstract

Human type 1 insulin-like growth factor receptor is a homodimeric receptor tyrosine kinase that signals into pathways directing normal cellular growth, differentiation and proliferation, with aberrant signalling implicated in cancer. Insulin-like growth factor binding is understood to relax conformational restraints within the homodimer, initiating transphosphorylation of the tyrosine kinase domains. However, no three-dimensional structures exist for the receptor ectodomain to inform atomic-level understanding of these events. Here, we present crystal structures of the ectodomain in apo form and in complex with insulin-like growth factor I, the latter obtained by crystal soaking. These structures not only provide a wealth of detail of the growth factor interaction with the receptor’s primary ligand-binding site but also indicate that ligand binding separates receptor domains by a mechanism of induced fit. Our findings are of importance to the design of agents targeting IGF-1R and its partner protein, the human insulin receptor.

## Abstract (web summary)

The human type 1 insulin-like growth factor receptor (IGF-1R) is important for normal human growth and development. Here, the authors present the crystal structures of the IGF-1R ectodomain both in its apo form and in complex with its ligand insulin-like growth factor I and discuss the receptor activation mechanism.

## Introduction

The human type 1 insulin-like growth factor receptor (IGF-1R) is a homodimeric, disulphide-linked (αβ)_2_ receptor tyrosine kinase implicated in normal human growth and development^1^. Aberrant IGF-1R signalling is implicated in cancer proliferation and metastasis^2^ and IGF-1R has undergone extensive investigation as an anti-cancer target^3^. IGF-1R is closely related to the human insulin receptor (IR): their ectodomains share 53% sequence identity, their respective monomers can form functional heterodimers and both receptors can bind all three ligands within the family (insulin and the two insulin-like growth factors, IGF-I and IGF-II), albeit with varying affinities^4^. The bioavailability, activity and tissue distribution of the IGFs are controlled by a suite of six insulin-like growth factor binding proteins^5^, as well as (in the case of IGF-II) by the type 2 insulin-like growth factor receptor/cation-independent mannose-6-phosphate receptor^6^, a receptor unrelated to IGF-1R.

**Fig. 1:**
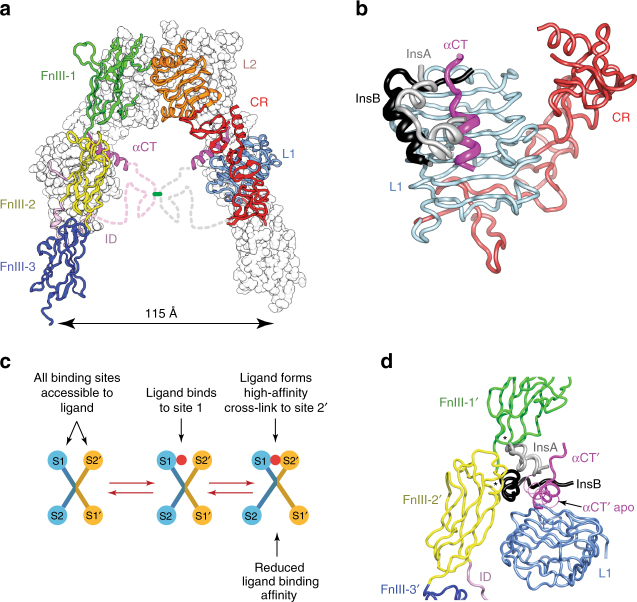
Structural biology of apo IRΔβ and insulin-bound μIR and the current model of ligand binding kinetics. **a** The Λ-shaped assembly of IRΔβ (PDB entry 4ZXB)^8^. Domain colours are L1 light blue, CR red, L2 orange, FnIII-1 green, FnIII-2 yellow, FnIII-3 dark blue, ID light magenta, αCT magenta. The foreground monomer is in ribbon representation, the background monomer in surface representation (apart from the ID element); dashed lines indicate disordered residues within the respective ID segments. **b** Human insulin (A chain grey, B chain black) bound to μIR (PDB entry 4OGA)^11^, coloured as in **a**. **c** Major pathway of ligand binding to IR and IGF-1R within the current kinetic model. S1, S2: site 1 and site 2 on one receptor monomer; S1΄, S2΄: site 1 and site 2 on the opposing receptor monomer. Red filled circle: ligand (i.e., IGF-I, IGF-II or insulin). **d** Steric overlap (asterisked) between insulin and the opposing fibronectin domain module of the structures depicted in **a** and **b** based on overlay of their common domain L1. αCT΄ is shown in both its apo conformation (thin magenta ribbon) and its insulin-complexed conformation (magenta ribbon) in order to illustrate its altered disposition upon insulin binding

Whereas no three-dimensional structures exist of the intact IGF-1R ectodomain, two IR-based crystal structures provide insight into how IGFs might bind to IGF-1R. The first is that of apo IRΔβ, an IR ectodomain-only construct that lacks a short, glycosylated segment near the receptor β-chain N terminus which is deemed superfluous to function^7,8^. IRΔβ is Λ-shaped (Fig. 1a), with the first leucine-rich repeat domain (L1), the cysteine-rich domain (CR) and second leucine-rich domain (L2) of each receptor monomer being juxtaposed against a linear arrangement of the first, second and third type III fibronectin domains of the opposing αβ monomer (FnIII-1΄, -2΄ and -3΄; the ΄ symbol denoting here and below entities from the alternate monomer). The insert domain (ID; an ~110-residue region within FnIII-2 and which contains the α/β cleavage site) lies predominantly within the interior of the Λ-shaped assembly. The C-terminal region of the α-chain component of ID΄ contains a segment (αCT΄) that assembles as an α helix on the central β sheet (L1-β_2_) of domain L1—together, these elements form site 1, the primary ligand-binding site^9^. The degree of sequence identity of IGF-1R and IR suggests that the IGF-1R ectodomain has a similar three-dimensional structure to that of IR. The second structure is that of insulin co-complexed with an isolated IR L1-CR module and exogenous αCT peptide (Fig. 1b)^10,11^; the latter receptor elements minimally reconstitute site 1^12^ and are together termed the insulin “microreceptor” (μIR)^11^. This structure reveals that both insulin and αCT undergo substantial conformational change upon site 1 engagement. Again, it is reasonable to assume, given the structural relationship between the ligands^13^, that the IGFs bind the primary binding site of IGF-1R in a fashion similar to that of insulin to μIR^14^. Indeed, mutagenesis of IGFs reveals a high degree of correspondence of their respective receptor-binding surfaces to those of insulin^15,16^.

Little is known about how either IGFs or insulin interact with the secondary binding site (site 2) of their cognate receptor, an event deemed essential for receptor activation^17^. The current kinetic model of ligand binding to IGF-1R and IR assumes that the apo receptor is in an “open” conformation with all four binding sites (1, 1΄, 2 and 2΄) exposed to incoming ligand. Incoming ligand engages first with site 1, and then forms a cross-link to site 2΄, located on the alternate monomer to that which contributes domain L1 to site 1 (Fig. 1c), with the resultant cross-link being of high affinity^18^. An immediate conundrum is that overlay of the above two IR−based structures indicates that insulin is sterically prevented from forming a site 1 complex with apo IRΔβ without displacement of the receptor L1-CR module away from domains FnIII-1΄ and FnIII-2΄ (Fig. 1d), i.e., the apo receptor structure is in a “closed” conformation. Resolution then requires either that (i) ligand binding itself separates domain L1 from the opposing FnIII΄ domain module, (ii) ligand binds to only a dynamically transient population of receptor conformations that already have these domains displaced from each other (i.e., “open” receptors) or (iii) the apo-IRΔβ structure does not reflect the ectodomain structure within apo holo-receptor. Biochemical and biophysical analyses of IGF-1R indicate that the separation of domain L1 from the opposing FnIII΄ domain modules is in fact integral to IGF-1R activation^19^. These analyses suggest further that, in the ligand-free state, the receptor transmembrane domains are held apart by the Λ-shaped assembly of the ectodomain, but ligand-induced separation of domain L1 from the FnIII΄ domain module then releases the conformational constraint on the latter, allowing the attached transmembrane (TM) helices to interact and autophosphorylation to occur^19^. Equivalent data do not exist for IR; however, there is indication that for IR, receptor activation may instead involve the separation (rather than coming together) of the transmembrane helices within the homodimer^20^.

To address these issues and gain understanding of the mechanism of ligand binding, we have determined crystal structures of apo- and IGF-1-bound forms of IGF-1RΔβ, the latter intriguingly obtained via crystal soaking. IGF-1RΔβ, like IRΔβ, is an ectodomain-only construct that lacks the likely disordered and non-functional segment near the N terminus of the receptor β chain^21^. These structures, refined using data to resolution of 3.0 Å and 3.27 Å, respectively, were both obtained as co-complexes with an antibody variable-domain module (Fv). Not only do our structures provide a wealth of atomic detail regarding IGF-1R and its interaction with IGF-I, but they also lead to new insights into the receptor activation mechanism, relevant to those seeking to design novel agents targeting IGF-1R and/or IR.

## Results

### Characterization of IGF-1RΔβ

Labelled-ligand competition binding assays show that IGF-I and IGF-II bind IGF-1RΔβ with half-maximal inhibitory concentration (IC_50_) values of 0.14 nM (0.12–0.17 nM) and 0.33 nM (0.23–0.48 nM), respectively, with the values in parentheses being the 95% confidence intervals (Supplementary Figure 1). These values align closely with those reported for IGFs (0.41 ± 0.1 nM and 0.88 ± 0.6 nM, respectively)^22^ in similar assays of an isolated IGF-1R ectodomain devoid of the “Δβ” modification, demonstrating that the modification does not affect ligand affinity.

### Structure of IGF-1RΔβ + Fv 24-60

**Fig. 2:**
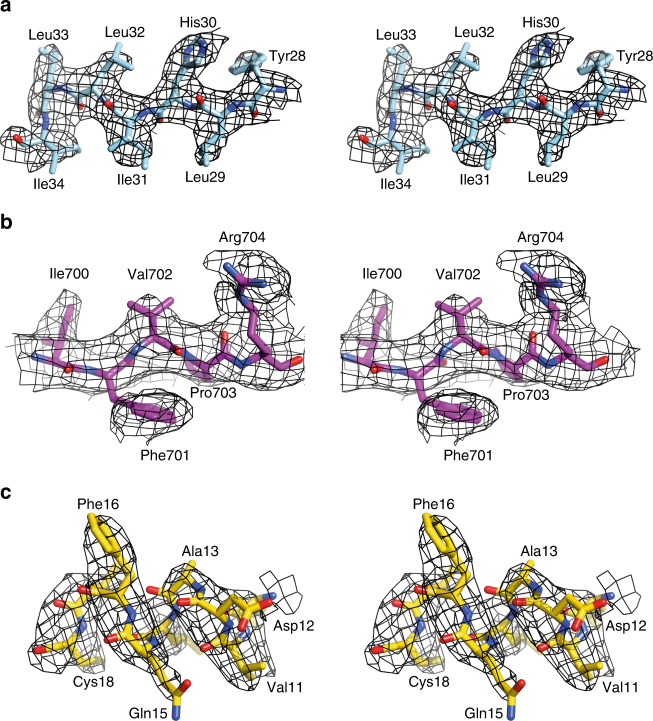
Stereo view of representative σ_A_-weighted (2*F*_o_*−F*_c_) difference electron density. **a** The σ_A_-weighted (2*F*_o_*−F*_c_) difference electron density in the vicinity of IGF-1RΔβ L1 domain residues 28–34 within the crystal of apo IGF-1RΔβ + Fv 24–60. The density is sharpened (*B*_sharp_ = −60 Å^2^) and displayed at a contour level of 1.7 σ (σ = root-mean-square deviation of the sharpened map). Density is shown only for volume within 2.0 Å of the atoms displayed. **b** The σ_A_-weighted (2*F*_o_*−F*_c_) difference electron density in the vicinity of IGF-1RΔβ αCT residues 700–704 within the crystal of apo IGF-1RΔβ + Fv 24–60. The density is sharpened (*B*_sharp_ = −60 Å^2^) and displayed at a contour level of 0.33 σ (σ = root-mean-square deviation of the sharpened map). Density is shown only for volume within 2.5 Å of the atoms displayed. **c** σ_A_-weighted (2*F*_o_*−F*_c_) difference electron density in the vicinity of IGF-I residues 11–18 within the crystal of the IGF-I-complexed IGF-1RΔβ + Fv 24–60. The density is sharpened (*B*_sharp_ = −60 Å^2^) and displayed at a contour level of 1.7 σ (where σ is the root-mean-square deviation of the sharpened map). Density is shown only for volume within 2.0 Å of the atoms displayed

The structure of the IGF-1R ectodomain was obtained by X-ray diffraction analysis of a crystal of IGF-1RΔβ in complex with the Fv module of the monoclonal antibody (mAb) 24–60^23^, the latter employed here as a crystallization chaperone^24^. The crystal used displayed diffraction to a maximum resolution of ~3.0 Å (albeit anisotropically; see Methods and Table 1). Within the crystallographic unit cell, the IGF-1RΔβ homodimer has twofold crystallographic symmetry and structure solution was by molecular replacement, employing as search objects the L1-CR and L2 fragments of IGF-1R (from the structure of the isolated L1-CR-L2 fragment of the receptor^25^) and homology models of Fv 24–60 and of IGF-1RΔβ FnIII-1,-2 and -3, the latter three based on their counterparts within IRΔβ^9^. The structure was refined using all data to a resolution of 3.0 Å; statistics are in Table 1 and representative difference electron density in Fig. 2a.

**Table 1:** X-ray diffraction data collection and refinement statistics^a^

	apo IGF-1RΔβ + Fv 24–60 (PDB 5U8R)	IGF-1RΔβ + Fv 24–60 + IGF-I (PDB 5U8Q)
*Data collection*		
Space group	P2_1_2_1_2	P2_1_2_1_2
Cell dimensions		
*a*, *b*, *c* (Å)	95.03, 201.73, 117.75	88.69, 197.66, 117.65
*α*,* β*, *γ* (°)	90, 90, 90	90, 90, 90
Resolution (Å)	50.00–3.00 (3.13–3.00)^b^	50–3.27 (3.46–3.27)
*R*_merge_	0.25 (3.47)	0.19 (2.07)
*I /* σ(*I*)	8.3 (0.6)	9.1 (0.9)
*CC*_1/2_	0.999 (0.551)	0.998 (0.775)
Completeness (%)	99.7 (99.5)	98.6 (92.5)
Redundancy	8.9 (7.2)	7.2 (6.7)
*B*_11_, *B*_22_, *B*_33_ (Å^2^)^c^	115.2, 117.6, 62.4	157.3, 128.4 71.8
*Refinement*		
Resolution (Å)	46.36–3.00	22.18–3.27
No. of reflections	45,805	32,110
*R*_work_/*R*_free_	0.256/0.285	0.260/0.303
No. of atoms		
Protein	8194	8537
Carbohydrate	112	148
Solvent	28	19
*B* factors (Å^2^)		
Protein	134	177
Carbohydrate	146	202
Solvent	125	178
R.m.s. deviations		
Bond lengths (Å)	0.002	0.002
Bond angles (°)	0.48	0.52

^a^ Each data set was collected from a single crystal

^b^ Values in parentheses are for highest-resolution shell. The resolution limit was set at being the maximum at which the *CC*_1/2_ statistic^43^ remained significant at the *P* = 0.001 level of significance

^c^ Maximum likelihood estimate of overall *B*_cart_, calculated using XTRIAGE within the PHENIX suite^49^

**Fig. 3:**
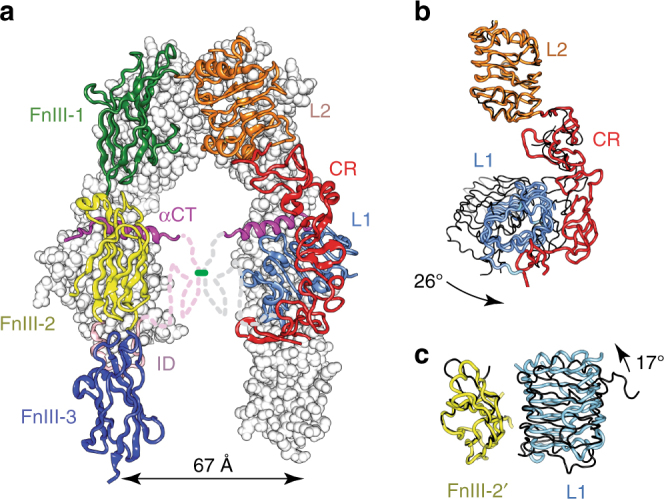
The crystal structure of apo IGF-1RΔβ. **a** The Π-shaped assembly of IGF-1RΔβ. Domain colours are L1 light blue, CR red, L2 orange, FnIII-1 green, FnIII-2 yellow, FnIII-3 dark blue, ID light magenta, αCT magenta. The foreground monomer is in ribbon representation, the background monomer in atomic sphere representation (apart from the ID element); dashed lines indicate disordered residues within the respective ID segments. **b** L1-CR-L2 module of IGF-1RΔβ (coloured as in **a**) overlaid onto that of IRΔβ (black) on the basis of corresponding residues within the L2 domain, showing the 26° difference in relative orientation of L1-CR and L2 in the two receptors. **c** L1/FnIII-2΄ pair of IGF-1RΔβ (coloured light blue and yellow, respectively) overlaid onto that of IRΔβ (black) on the basis of corresponding residues within domain FnIII-2΄, showing the 17° difference in relative orientation of L1 and FnIII-2΄ in the two receptors

The quaternary structure of IGF-1RΔβ (Fig. 3a) exhibits the same folded-over conformation as IRΔβ, with the locations of secondary structural elements and domain boundaries being closely similar to those of IRΔβ. No electron density is seen for ID residues 642–690; these residues contain the inter-monomer disulphide bond motif at Cys669-Cys670-Ala-671-Cys672. Electron density is also poorly defined for residues 509–516 within domain FnIII-1, this loop contains the inter-monomer disulphide bond at residue Cys514. The equivalent disulphide bond regions are also poorly defined in the structure of IRΔβ. In contrast, electron density for the α-chain to β-chain disulphide bond (linking Cys633 to Cys849) is well defined. *N*-linked glycan residues could be modelled convincingly at sites Asn21, Asn105, Asn504, Asn577, Asn610 and Asn883. Of the remaining potential *N*-linked sites within IGF-1RΔβ, electron density features were seen extending from the respective side chains of Asn214, Asn284, Asn387, Asn408, Asn870 and (possibly) Asn592, but these were left unmodelled due to lack of adequate order. Some electron density was present in the vicinity of the side chain of Asn72—it is unknown whether this site is glycosylated in IGF-1R, though mass spectrometry has revealed that its counterpart (Asn78) in IR is devoid of glycosylation^26^. The disposition of the αCT΄ helix (residues 684–697) upon the surface of domain L1 is also closely similar to that within the structures of apo IRΔβ^9^ and apo μIR^27^. The resolution of the current structure was sufficient to avoid ambiguities in the strand register within domains FnIII-1 and FnIII-2, an issue which bedevilled the original structure determination of IRΔβ^8^. We note further that, within the crystal lattice, substantial solvent volume exists in the vicinity of the first modelled residue (Glu744) of the β chain of IGF-1RΔβ, indicating that the observed structure is not in conflict with that which could be adopted by the intact IGF-1R ectodomain (i.e., one devoid of the “Δβ” mutation/deletion).

The crystallization chaperone, Fv 24–60, is seen attached to domain CR, consistent with the epitope reported for its parent mAb^23^. Further detail of its epitope is provided below. Inspection of the unit cell reveals that Fv 24–60 mediates the majority of lattice contacts, consistent with its use to overcome the hindrance to crystallization posed by the *N*-linked glycans. The mAb 24–60 is reported to reduce by 90% the affinity of IGF-I binding to a cell-bound receptor and to a lesser degree the affinity of binding to a soluble receptor^23^. Here, the Fv module does not interact sterically with any receptor components beyond its epitope, suggesting that relative disposition of domains with the ectodomain has not been modulated by Fv attachment per se (see below for further discussion of the likely cause of ligand affinity reduction).

Despite the above similarities, two salient differences emerge between the structures of IGF-1RΔβ and IRΔβ. First, the sites of membrane entry (i.e., the respective C termini of domains FnIII-3 and FnIII-3΄) are substantially closer together in IGF-1RΔβ (~67 Å) than in IRΔβ (~115 Å), i.e., the overall shape of IGF-1RΔβ is more “closed” (Fig. 3a) than that of IRΔβ (Fig. 1a). The altered spacing reflects cumulative differences in the relative orientations of consecutive domains within the receptor monomers, with the largest being a 26° difference between the two receptors in the relative orientation of the L1-CR module with respect to its downstream domain L2 (Fig. 3b). These intra-monomer differences in domain orientation accumulate to provide a 17° difference between the two receptors in the alignment of domain L1 of one monomer with respect to domain FnIII-2΄ of the adjacent monomer (Fig. 3c).

**Fig. 4:**
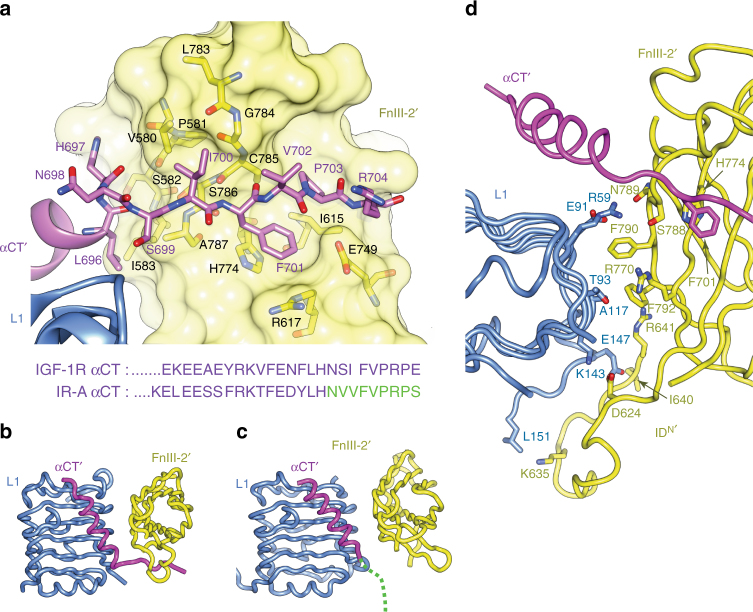
Configuration of αCT΄ and domains L1 and FnIII-2΄ of apo IGF-1RΔβ. **a** Ordering of the C-terminal region of the apo IGF-1RΔβ αCT΄ segment upon the surface of the adjacent domain FnIII-2΄. Inset below is a sequence alignment of residues at the C terminus of the respective α chains of IGF-1RΔβ and IRΔβ; residues in green are disordered in the crystal structure of the IRΔβ^8^. Note that the αCT segment of IRΔβ is that of the A isoform of the receptor^7^. **b** Association of the αCT΄ segment with domains L1 and FnIII-2΄ within the crystal structure of apo IGF-1RΔβ. **c** Association of the αCT΄ segment with domain L1 alone within the crystal structure of apo IRΔβ. Green dashed line represents the disordered C-terminal region of the α΄ chain of IRΔβ. The view direction in **b** and **c** is equivalent with respect to the domain L1. **d** Interaction between domains L1 and FnIII-2΄ within apo IGF-1RΔβ

The second difference lies in the IGF-1RΔβ αCT΄ residues 698–704, which are located C terminal to the αCT΄ helix (residues 684–696). These residues are well resolved (Fig. 2b) and pack against the surface of the adjacent domain FnIII-2΄ (Fig. 4a, b). In contrast, in the structure of apo IRΔβ, the αCT΄ segment is entirely disordered C terminal to His710 (=IGF-1RΔβ His697), with the axis of the IR αCT΄ helix (residues 694–710) being directed away from IR domain FnIII-2΄ (Fig. 4c). This difference correlates with the differing alignments in the two receptors of domain L1 with respect to the cognate and adjacent domain FnIII-2΄ (Fig. 3c).

Of particular interest is the detail of the interface between domain L1 and domain FnIII-2΄ within the homodimer, as separation of these domains is implicated in receptor activation^19^. Here, the observed interface is sparse (Fig. 4d), burying only ~896 Å^2^ of molecular surface from solvent and with low shape correlation (*S*_c_ = 0.47)^28^. The nature of the interface thus appears consistent with one that is capable of in vivo disassembly. Part of the interface includes residues from the N-terminal segment (denoted ID^N΄^) of the ID΄ as they fold beneath domain L1. Change in solvent accessibility of this segment upon ligand binding has been detected in hydrogen/deuterium exchange experiments^29^.

### Structure of IGF-1RΔβ + Fv 24–60 + IGF-I

Crystals of the complex of IGF-I with the Fv-bound IGF-1RΔβ were obtained by soaking IGF-I directly into crystals of the receptor ectodomain/Fv complex. Incorporation by soaking of IGF-I into the apo crystals is remarkable, and is presumably facilitated by the crystal’s high solvent content (~75%). Soaking resulted in altered unit cell dimensions (Δ*a* = −6.3 Å, Δ*b* = −2.3 Å, Δ*c* = −0.3 Å) without change in space group. We are not aware of any other instance where such a large moiety as IGF-I (molecular weight = 7.7 kDa) has been incorporated into crystals by soaking. Despite cracking, only limited loss of diffraction resolution occurred (compared to the resolution typical in our hands of the parent crystals), but anisotropy persisted. Diffraction data were processed to 3.27 Å resolution (Table 1). The structure was solved by molecular replacement, using the domains of the apo IGF-1RΔβ + Fv 24–60 structure as search objects (see Methods). Difference maps revealed IGF-I bound to the single site 1 within the asymmetric unit, in a fashion effectively identical to that seen in liganded-μIR structures^10,27^, allowing its ready incorporation into the atomic model. Refinement statistics are in Table 1 and representative difference electron density in Fig. 2c.

**Fig. 5:**
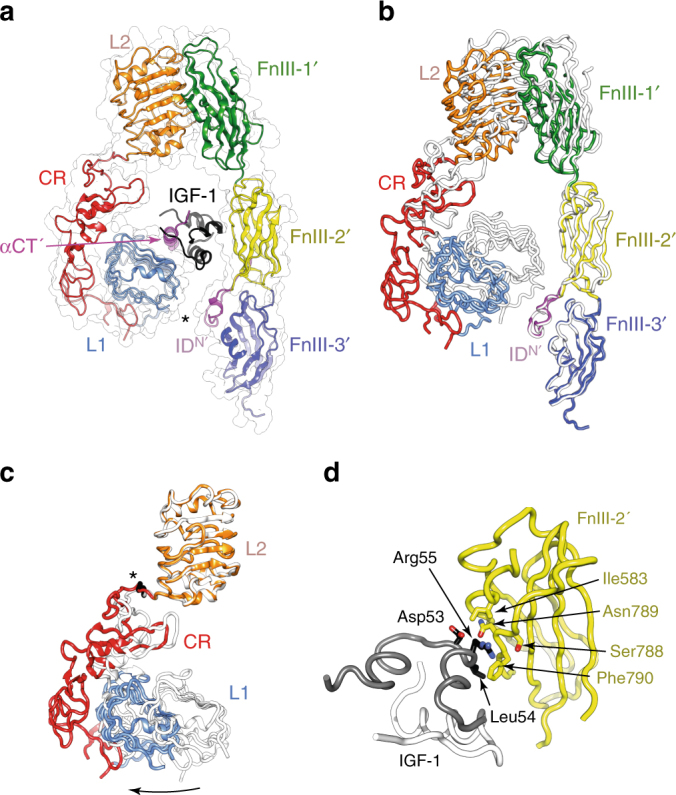
Mode of IGF-I binding to IGF-1RΔβ. **a** Bridge formed by IGF-I (black) between the site 1 components L1 and αCT΄ and FnIII-2΄, showing separation (asterisked) of the L1-CR module away from FnIII-2΄ and ID^N΄^. **b** Overlay of one “leg” of the IGF-1RΔβ homodimer in its IGF-1-bound form (coloured ribbon) onto the corresponding domains of the apo IGF-1RΔβ homodimer (white ribbon). Alignment is based on domains FnIII-2΄ and FnIII-3΄. **c** Overlay (via L2) of the L1-CR-L2 module of IGF-1-bound IGF-1RΔβ (coloured ribbon) onto that of apo IGF-1RΔβ (white ribbon). Pro297, the hinge point, is in black and asterisked. **d** Interaction between the site-1-bound IGF-I (A domain black, B domain white) and FnIII-2΄

Analysis of the structure reveals that IGF-I binding is accompanied by a separation of the IGF-1-bound L1-CR module away from domain FnIII-2΄ (Fig. 5a, b), this displacement being effected largely by a “hinge” motion close to the junction between domains CR and L2 (Fig. 5c). Conformational variation at this junction has been seen across extant structures of the IR ectodomain and its fragments^7,10,30^. The site-1-bound IGF-I also interacts with domain FnIII-2΄, the interface involving residues Ile583, Ser788, Asn789 and Phe790 of the receptor and residues Asp53, Leu54 and Arg55 of IGF-I. This interface is remarkably sparse (Fig. 5d) and hence, in our judgement, does not likely reflect the site 2 interaction—indeed, of the IGF-I residues involved in its formation, only Leu54 is deemed on the basis of alanine scanning mutagenesis^15,16^ to engage site 2. Whereas at physiological concentrations of ligand, only one IGF molecule is anticipated to be bound to the cell-surface expressed receptor^18^, the presence here of two IGF-I molecules within the homodimer is likely a consequence of the supra-physiological concentration of IGF-I soaking and its subsequent binding within the crystal.

*N*-linked glycan could be convincingly modelled at sites Asn21, Asn105, Asn504, Ans577, Asn870 and Asn883, with weaker density suggestive of carbohydrate (and left unmodelled) seen extending from the side chains of sites Asn214, Asn284, Asn408, Asn592 and Asn610. No indication of glycan was apparent at site Asn72. None of the *N*-linked glycan (modelled or otherwise) appeared to be in the immediate vicinity of the bound IGF-I. Again, substantial solvent volume is apparent in the vicinity of the first modelled residue (Tyr745) of the β chain of IGF-1RΔβ, indicating that the observed structure is not in conflict with that which could be adopted by an intact IGF-1R ectodomain devoid of the Δβ modification.

### The mode of engagement of IGF-I with site 1

Conformational changes are seen in both the αCT΄ helix and IGF-I upon IGF-I binding to site 1 of IGF-1RΔβ. These changes largely mimic those seen in the structure of IGF-I bound to the IR L1-CR+IGF-1R αCT hybrid-microreceptor complex^27^ and in the structure of insulin bound to the μIR^10,11^. In particular, they include remodelling of the αCT΄ helix on the L1-β_2_ surface and a folding out of the C-terminal region of the B domain of IGF-I away from the hormone core in order to allow its engagement by key residues within the αCT΄ helix.

**Fig. 6:**
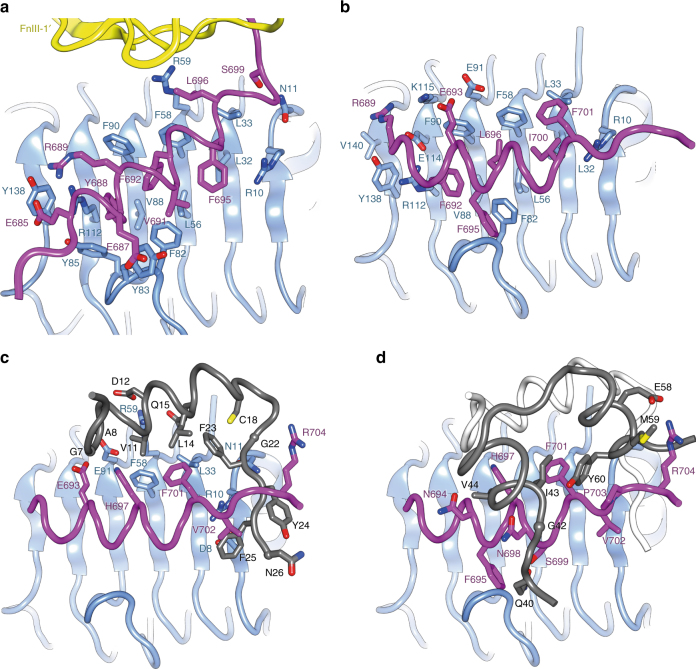
Dissection of the interaction of IGF-I with binding site 1 of IGF-1R. **a** Conformation of L1-β_2_ (light blue) and αCT΄ (magenta) in the apo IGF-1RΔβ structure, compared with **b**, its conformation in the IGF-I liganded IGF-1RΔβ structure. **c** Interaction between B domain of IGF-I (black) and the IGF-1R site 1 elements of L1-β_2_ (light blue) and αCT΄ (magenta). The A domain of IGF-I (located in the foreground) is omitted for clarity. **d** Interaction between A domain of IGF-I (black) and the IGF-1R site 1 element αCT΄ (magenta); no interaction is observed between the A domain and L1-β_2_ (light blue). The IGF-I B domain is in white

Details are as follows. In the apo IGF-1RΔβ structure, the αCT΄ helix spans residues 684–696 and engages (via the side chains of residues Tyr688, Phe692 and Phe695) a hydrophobic trough formed by the side chains of residues Leu32, Leu56, Phe58, Phe82, Tyr83, Val88 and Phe90 on the surface of L1-β_2_ (Fig. 6a). A potential salt bridge occurs between the side chains of αCT΄ residue Glu685 and L1 residue Arg112. Residues 681–683 are in an extended conformation N terminal to the αCT΄ helix, while C terminal to the helix, residues 697–704 order on the surface of the adjacent FnIII-2΄ domain (see above). Upon IGF-I binding, αCT΄ remodels, its helix now spanning residues 688–701, i.e., αCT΄ unwinds by one turn at its N-terminal end and extends by one turn at its C-terminal end (Fig. 6b). Concomitantly, the helix re-orients to lie approximately perpendicular to the direction of the L1-β_2_ strands and to engage the hydrophobic L1-β_2_ surface via the side chains of residues Phe692, Phe695, Leu696, Ile700 and Phe701 (Fig. 6b).

IGF-I, upon engagement with IGF-1RΔβ site 1, is also seen to undergo a conformational change similar to that seen in the hybrid microreceptor complex of IGF-I with IR L1-CR + IGF-1R αCT (and analogously in the microreceptor complex of insulin with IR L1-CR + IR αCT). IGF-I residues Tyr24 and Phe25 are displaced from the core of the growth factor, with the side chain of IGF-1R αCT΄ residue Phe701 now locating into volume originally occupied by the side chain of IGF-I Phe25 (Fig. 6c). The side chain of IGF-I Phe23 undergoes rotameric re-arrangement to bury in a largely hydrophobic pocket formed by the side chains of domain L1 residues Asn11, Leu33, αCT΄ residue Phe701 and IGF-I residues Leu14, Gln15 and Cys18 (Fig. 6c), as well as by the main chain atoms of IGF-I residue Tyr60. The side chain of IGF-I residue Tyr24 interacts with the side chains of αCT΄ residues Val702 and Arg704 and with that of IGF-I residue Asn26. The side chain of IGF-I residue Phe25 stacks against those of domain L1 residues Asp8 and Arg10 and αCT΄ residue Val702. (Fig. 6c). No interpretable electron density is apparent for IGF-I B-domain residues 27–30 nor C-domain residues 31–38, the only C-domain residues in interpretable density thus being residues Pro39, Gln40 and Thr41. Of these latter residues, only Gln40 interacts here with the receptor, via αCT΄ residues Phe695 and Ser699 (Fig. 6d). The absence of density for IGF-I residues 27–38 is important, as Tyr31, Arg36 and Arg37 have been shown by site-directed mutagenesis to be critical contacts for high-affinity IGF-I binding (reviewed in Denley et al.^31^). In particular, grafting the IGF-I C domain into the insulin molecule raises the affinity of insulin for IGF-1R to 19–28% of that of IGF-I^32^. The absence here of a visible interaction between elements of the C domain and IGF-1R may be caused by the attachment of Fv 24–60 (see below). While the absence of density for the IGF-I C domain does not formally resolve the issue as to whether or not the αCT peptide “threads” through the loop formed by the C domain and the helical core of IGF-I^27^, residual electron density between IGF-I residues 26 and 39 in the vicinity of IGF-1R domain CR suggests that such threading occurs. Contacts between IGF-I and the site 1 elements of the receptor are summarized in Supplementary Table 1. Also included in Supplementary Table 1 are literature-derived data regarding the effect of mutations on IGF-I binding to IGF-1R—these data indicate that many of the site-1-engaging residues of IGF-I are critical to the interaction. In particular, IGF-I residue Val44 (Fig. 6d) is a critical site 1 contact; mutation of this residue to (the larger) methionine causes dwarfism^31^. Mutation to (the larger) leucine at the equivalent ValA3 position in insulin Wakayama causes diabetes^33^.

### Inhibitory nature of mAb 24–60

**Fig. 7:**
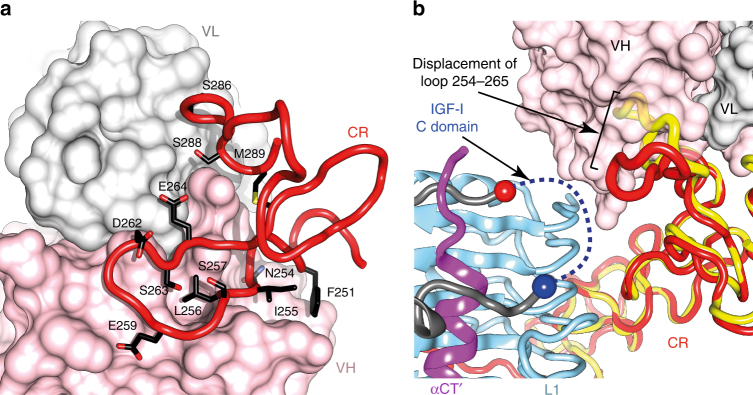
Interaction of Fv 24–60 with IGF-1RΔβ. **a** Interaction of residues of domain CR of IGF-1RΔβ (red) with Fv 24–60 (variable heavy chain domain pink; variable light chain domain light grey). **b** Displacement by Fv 24–60 of the peptide loop formed by IGF-1RΔβ CR residues 254–265 towards the IGF-I binding site. The yellow ribbon is that of IGF-1R domain CR within the crystal structure of the isolated L1-CR-L2 fragment of IGF-1R obtained in the absence of attached Fv (PDB entry 1IGR^25^), overlaid onto that of the IGF-I-bound IGF-1RΔβ structure (red) on the basis of common domain L1. Red sphere: IGF-I Asn26; blue sphere: IGF-I Pro39. The connecting IGF-I domain C residues 27–38 (indicated putatively by a blue dashed line) are disordered

The mAb 24–60 has been shown to inhibit IGF-I (but not IGF-II) binding to IGF-1R by up to 90%^22,23^. The Fv 24–60 epitope is seen here to comprise primarily the residue 254–265 loop of domain CR (Fig. 7a). This loop contains a number of acidic residues implicated in IGF affinity and selectivity, potentially through interaction with basic residues within the C domain of IGFs^30,34,35^. The residue 254–265 loop has an effectively identical conformation in the two structures presented here, but its location differs significantly from that in the isolated (and Fv-free) L1-CR-L2 fragment of IGF-1R^25^, being displaced here towards the volume that must implicitly be occupied by the (disordered) C domain of IGF-I (Fig. 7b). We hypothesize therefore that the inhibitory nature of mAb 24–60 arises allosterically from steric interference of the displaced 254–265 loop with the C domain of the IGF-I. Supporting this contention is the fact that mAb 24–60 does not affect IGF-II binding to IGF-1R^22^—the salient difference between IGF-II and IGF-I is the four-residue shorter C domain of IGF-II. To test this hypothesis, we determined, using isothermal titration calorimetry (ITC; Supplementary Figure 2), the affinity of (a) IGF-I for IGF-1RΔβ: *K*_d_ = 39 ± 8 nM (*n* = 4), (b) IGF-I for IGF-1RΔβ pre-complexed with Fv 24–60: *K*_d_ = 2.4 ± 0.5 μM (*n* = 2) and (c) IGF-I CII (an IGF-I chimera that contains the shorter C-domain of IGF-II^36^) for IGF-1RΔβ pre-complexed with Fv 24–60: *K*_d_ = 32 ± 8 nM (*n* = 2), i.e., replacement of the IGF-I C domain by that of IGF-II abrogates inhibition by Fv 24–60. These data are consistent with the inhibitory nature of mAb 24–60 with respect to IGF-I binding arising from a compromising interaction between the antibody-conjugated receptor and the C domain of IGF-I. We note that the *K*_d_ value for IGF-I binding to IGF-1RΔβ is lower (i.e., numerically greater) than the IC_50_ value reported in the competition binding assay mentioned above; this difference is likely associated with the much higher receptor concentration in the ITC measurements. ITC-derived *K*_d_ values for IGF-1R have not been reported prior in the literature.

### Mutational analysis of residues within FnIII-2΄

Our structures suggest that a number of residues within domain FnIII-2΄ may play a role in receptor functioning. These residues include (a) His774, which interacts with αCT΄ residue Phe701 within the apo ectodomain structure (Fig. 4d), (b) Ser788, Asn789 and Phe790, which interact with IGF-I within the ligand-complexed ectodomain structure (Fig. 5d), and (c) Phe792, which, together with Phe790, interacts with domain L1 within the apo ectodomain structure (Fig. 4d). To investigate the role of these five residues, we transiently transfected IGF-1R knockout fibroblasts (R^-^ cells) with individual plasmids encoding IGF-1R genes each carrying alanine mutation at one of the five above sites. The double-alanine mutant Phe790Ala/Phe792Ala was also tested. All constructs led to the expression of mutant IGF-1R (Supplementary Figures 3a, 3b and 4), apart from the Phe790Ala/Phe792Ala mutant, which was poorly expressed compared to the wild-type receptor and thus not considered further. None of the five mutant receptors expressed displayed an affinity for IGF-I significantly different from that of wild-type receptor (Supplementary Table 2 and Supplementary Figure 3c). The lack of difference in affinity of these mutants for IGF-I with respect to that of the wild-type receptor supports our above assessment that the interaction of Ser788, Asn789 and Phe790 with IGF-I observed in the soaked crystals is likely non-physiological (Fig. 4d). The relative activation of the mutant cells by IGF-I was also determined by measuring the relative degree of phosphorylation of the mutant IGF-1R residues Tyr1158, Tyr1162 and Tyr1163. There was no difference in the basal activation of all IGF-1R mutants and, upon IGF-I stimulation, the Ser788Ala, Asn789Ala and Phe792Ala mutants were activated to the same level as wild-type IGF-1R (Supplementary Figures 3b, 3d and 4). These data suggest that these residues are not significantly involved in ligand binding (in the case of Ser788 and Asn789) or in stabilizing the association between L1 and FnIII-2΄ (in the case of Phe792). Interestingly, His774Ala and Phe790Ala mutant receptors did not respond as well to IGF-I stimulation as the wild-type IGF-1R (Supplementary Figures 3b, 3d and 4). His774 appears to play a role in stabilizing the interaction of the αCT΄ C-terminal segment with domain FnIII-2΄ and Phe790 in stabilizing the interaction of domain L1 with domain FnIII-2΄ interaction in the apo receptor (Fig. 4d), suggesting in turn that these structural motifs may play a role in ligand-mediated receptor activation.

## Discussion

The structures presented here provide three-dimensional views of the IGF-1R ectodomain homodimer in apo and liganded form. The apo structure demonstrates that the IGF-1R apo ectodomain is similar in three-dimensional structure to that of IR, aligning with the ability of the respective receptor monomers to form functional hybrid receptors^37^. The source of the differing distances in IGF-1RΔβ and IRΔβ of their β-chain C termini is unclear: it may simply be a consequence of flexibility at the inter-domain boundaries of one or both receptors and their packing into different respective crystal lattices. However, if IGF-1R and IR do have a different mode of activation (i.e., intra-homodimer TM domain interaction^19^ vs intra-homodimer TM domain separation^20^), then the disparate distances apart of the TM domains of these two receptors may be associated with these differing modes. Likewise, the functional implications (if any) of the disparate arrangement of the C-terminal residues of the αCT΄ segment is unknown. We speculate that it may reflect a difference in the way the respective αCT΄ segments engage ligand: in the case of IGFs, the αCT΄ segment likely “threads” through the loop formed by the IGF C domain and the growth factor core, whereas such a topological requirement does not exist for site 1 engagement of the two-chain insulin molecule. Nevertheless, the IGF-1 complexed structure demonstrates that the mode of IGF-I engagement with the site 1 components (i.e., domain L1 and αCT΄) of the receptor is closely similar to that of insulin’s engagement with the corresponding components of IR^10,11^, again aligning with the ability of the receptors to bind each other’s ligand(s)^37^.

Together, the structures demonstrate that IGF-I binding obligates a separation of IGF-1R domain L1 away from the FnIII domain module of the adjacent monomer^19^. The manner in which this has occurred within the apo crystals raises the interesting issue as to what extent it reflects binding in vivo. The fact that binding can occur within the crystal suggests that the ligand binding process is one of induced fit that results not only in conformational change in the ligand and the receptor site 1 elements, but also in the concomitant separation of domain L1 from the adjacent FnIII΄ domains. Such induced fit must by its nature involve initial (likely metastable) engagement of the IGF-1 with structural elements of the receptor ligand binding cavity, the latter being in the form visualized in the apo receptor structure. The existence of such a ligand/receptor pre-complex that, upon relaxation, directs the receptor into an “open” conformation has not been considered prior in the literature. It is thus opportune to ask whether such binding is compatible with the extant kinetic data for the receptor. IGF-1R and IR exhibit complex kinetics, characterized by a curvilinear Scatchard plot and negative cooperativity. The latter is best exemplified by the accelerated dissociation of a pre-bound tracer (e.g., I^125^-labelled ligand) in the presence of unlabelled (“cold”) ligand under conditions of “infinite” dilution that preclude tracer rebinding^38^. These observations can be explained by the so-called harmonic oscillator (HO) model^18^, which assumes that the apo receptor exists in a continuum of energetic states that can be modelled as arising from harmonic oscillation of the receptor domains. The majority (~95%) of these conformations, under physiological conditions, are postulated to be “open”, i.e., all four sites (1, 1΄, 2 and 2΄) are exposed to incoming ligand (Fig. 1c). Within the HO model, conformational oscillation of the open receptor results either (i) in the presence of ligand, a ligand cross-link to site 2΄ (Fig. 1c), or (ii) in the absence of ligand, a low level of constitutive activity. However, while the HO model provides an accurate fit to the kinetic data, it cannot readily be mapped to the structures of apo IGF-1RΔβ or apo IRΔβ, as in these structures, sites 1 and 1΄ (and possibly also sites 2 and 2΄) are partly occluded from the incoming ligand (Fig. 1d). Two resolutions are proposed. The first is to assume that in vivo the receptor oscillations are such that 95% of receptor conformations are “closed” (i.e., inactive), with the incoming ligand being able to access site 1 only within the 5% of receptor conformations that are “open” (i.e., the inverse conformation percentages to those in the HO model). The second resolution is that physiological ligand binding occurs by a process similar to that observed in the crystal, i.e., that ligand binds to the “closed” receptor by a process of induced fit that concomitantly results in separation of domains L1 and FnIII-2΄. These models are not mutually exclusive and both may occur under physiological conditions. We note that regardless of the mode of binding, no more than one mole equivalent of ligand is expected to bind to the receptor (αβ)_2_ homodimer at physiological ligand concentrations, given that both IGF-1R and IR display negative cooperativity.

**Fig. 8:**
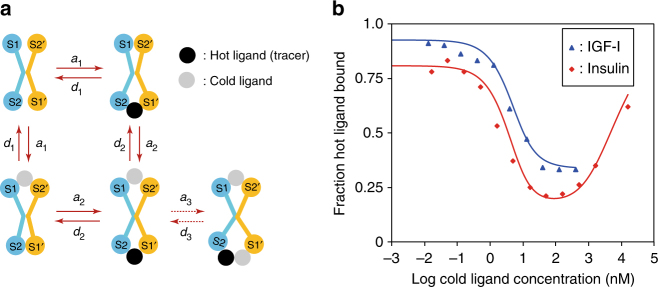
Induced fit binding of ligand to IGF-1R and IR. **a** Proposed kinetic scheme. S1, S2: site 1 and site 2 on one receptor monomer; S1΄, S2΄: site 1 and site 2 on the opposing receptor monomer. Black filled circle: hot ligand (i.e., IGF-I, IGF-II or insulin). Grey circle: cold ligand (i.e., IGF-I, IGF-II or insulin). *a*_1_ and *d*_1_: ligand association and dissociation rate constants for the high-affinity site. *a*_2_ and *d*_2_: ligand association and dissociation rate constants for doubly liganded, symmetrical receptor conformation. *a*_3_ and *d*_3_: association and dissociation rate constant for binding of a third insulin molecule (IR only, not applicable to IGF-1R). **b** Plot for accelerated dissociation of a pre-bound tracer-labelled ligand by cold (unlabelled) ligand. The dissociation time was 20 min. The experimental data were as described previously (reproduced in Supplementary Table 4)^18^ and are shown as blue triangles for IGF-1 and red diamonds for insulin, with the fit of the induced-fit model to these data shown as lines (IGF-I blue; insulin red)

However, the induced fit mechanism requires reformulation of the kinetic model. We have thus tested whether such reformulation agrees with the receptor binding and negative cooperativity data (see Methods). Inclusion of a doubly liganded, symmetrical receptor conformation under appropriate experimental conditions allows substantial simplification of the ligand binding scheme compared to that of the HO model (Fig. 8a, with detailed description provided in the Methods section). If rate constants are chosen to reflect a high-affinity site for ligand of *K*_d_ ≈ 0.2 nM and a low-affinity site of *K*_d_ ≈ 6 nM (i.e., approximately those values derived from the HO model analysis), with insulin having an additional binding site with a *K*_d_ ≈ 1000 nM, then simulation with these values is seen to yield good agreement with the experimental negative cooperativity data for both receptors (Fig. 8b).

We note that if physiological IGF-I binding to IGF-1R is indeed by a process of induced fit, then a corollary is that certain IGF-I residues may interact only transiently with receptor, i.e., during formation of the pre-complex, and it thus cannot be ruled out that some (even all) of the IGF-I residues currently understood to interact with site 2΄ (i.e., IGF-I residues Glu9, Asp12, Phe16, Leu54 and Glu58)^15^ may fall into this category. Such interactions would enhance the ligand on-rate to site 1 and contribute to high affinity.

In summary, our structure provides the first view of IGF-I in complex with site 1 of its receptor and the serendipitous way in which it was obtained by in situ crystal soaking has led us to propose a previously unconsidered mechanism of receptor activation. While the HO model remains a valid and general conceptual model (and was indeed the first concerted allosteric model able to incorporate negative cooperativity arising from ligand-stabilized asymmetry and bivalent cross-linking), its mathematical formalism has here been adapted and mapped onto the structural detail that has emerged for IGF-1R and for IR. The next challenge in the structural biology of this receptor family will be to understand the pathway by which the final ligand complex is formed and how it enables the intricate conformational change that directs the receptor to its final, activated state.

## Methods

### Expression and purification of IGF-1RΔβ

A CHO Lec8 cell line stably expressing IGF-1RΔβ (a construct of the human IGF-1R ectodomain comprising residues 1–905 but with the highly glycosylated segment (residues 718–741) near the N terminus of the β chain replaced by the quadruplet AGNN) was originally obtained from CSIRO (Parkville, Australia) by the corresponding author’s laboratory^21^. Cells were thawed into Dulbecco's modified Eagle's medium F12 + GlutaMAX medium (Life Technologies) containing 10 μg mL^−1^ puromycin (Life Technologies) plus 10% fetal bovine serum (Life Technologies) and expanded by passaging several times in T150 tissue culture flasks at 37 °C/5% CO_2_. Cells from the T150 flasks were then used to seed 850 cm^2^ roller bottles (Corning, Sigma-Aldrich); these were grown at 37 °C for 21 days but in the absence of CO_2_.

IGF-1RΔβ was recovered from harvested media by IGF-I affinity chromatography on a column of immobilized LONG-R3-IGF-I (GroPep Bioreagents; Australia) and then further purified by size-exclusion chromatography (SEC) as follows: the LONG-R3-IGF-1 affinity column was prepared by covalently binding 40 mg of media grade LONG-R3-IGF-I (GroPep Bioreagents; Australia) to 50 mL Mini-Leak Medium Agarose (Kem-En-Tec; Denmark) as per the manufacturer’s instructions. Typically, 10 L conditioned medium containing IGF-1RΔβ was pumped through a 50 mL column of Sepharose CL-4B (Sigma-Aldrich) to remove non-specifically binding material and then a 50 mL affinity column at 4 °C, followed by extensive column washing to remove unbound protein. Both columns were equilibrated with Tris-buffered saline (25 mM Tris-HCl, 137 mM NaCl, 2.7 mM KCl, pH 8.0) containing 0.02% sodium azide (TBSA). The affinity column was eluted with 0.4 M NaCl, 0.2 M tri-sodium citrate adjusted to pH 5.0 with HCl and also containing 0.02% sodium azide. Eluate was collected into 3 M Tris-HCl buffer, pH 8.5, to immediately adjust the pH to about 8 and 0.1 mM phenylmethylsulfonyl fluoride was added. Eluate was concentrated by stirred-cell ultrafiltration on a 30 kDa cut-off membrane (Amicon) and further purified by SEC on a Superdex S200 26/60 column (GE Healthcare Lifesciences) equilibrated with TBSA.

### IGF competition binding assay

The integrity of the IGF-1RΔβ protein product was confirmed by measuring its affinity for IGF-I and IGF-II (GroPep Bioreagents; Australia) within a europium-labelled IGF-I competition binding assay (Supplementary Figure 1)^22,36^. Wells of microtiter plates (Greiner Lumitrac 600) were coated with anti-IGF-1R monoclonal antibody 24–31^23^ (a gift from Professor K. Siddle) employing 250 ng per well in bicarbonate buffer, pH 9.2, and blocked with 0.5% bovine serum albumin in 20 mM Tris pH 7.4, 150 mM NaCl, and 0.1% (v/v) Tween-20 (TBST) for 2 h at room temperature. Purified IGF-1RΔβ (0.5 μg per well in TBST) or lysates of cells expressing IGF-1R mutants (100 µl) were added to each well and incubated overnight at 4 °C.

Receptor-grade human IGF-I (GroPep Bioreagents, Australia) was europium labelled as per the manufacturer’s instructions (DELFIA Europium-labelling kit; Perkin Elmer). Briefly, 0.43 mM IGF-I was incubated with 2 mM labelling reagent in a 30 μl reaction (0.1 M Na_2_CO_3_, pH 8.5) at 4 °C for 2 days. The reaction was terminated with 0.05 M Tris-HCl, 0.15 M NaCl (pH 7.5), and unbound europium was removed by Superdex 75 SEC (Amersham Pharmacia Biotech; Sweden) in the termination buffer. Approximately 3 × 10^6^ fluorescent counts of europium-labelled IGF-I were added to each well along with increasing concentrations of unlabelled IGF-I or IGF-II (GroPep Bioreagents; Australia) in a final volume of 100 μl and incubated for 16 h at 4 °C. Wells were washed four times with TBST, followed by the addition of 100 µl per well DELFIA enhancement solution (PerkinElmer Life Sciences). After 10 min, time-resolved fluorescence was measured using 340 nm excitation and 612 nm emission filters with a Victor X4 2030 Multilabel Plate Reader (Perkin Elmer). Nine technical replicates were performed for the IGF-I binding assay and six for the IGF-II binding assay. IC_50_ values were calculated by curve fitting with a one-site competition model using Prism 7.0 (GraphPad, USA).

### Expression and purification of Fv 24–60

The sequences of the variable heavy and variable light chain components of the mAb 24–60^23^ were determined under contract (Genscript) from amplified mRNA extracted from the hybridoma cell line (a gift of Professor K. Siddle, University of Cambridge). Synthetic DNA encoding a single-chain (sc) version of Fv 24–60 was inserted between the *Kpn*I and *Xho*I sites of the vector pgpHFT^39^, an in-house modified version of the vector pTriEx2 (Novagen; EMD Millipore), with the single-chain Fv sequence comprising residues 1–118 of the mAb heavy chain, followed by a linker segment of sequence ENLYFQGGGGGGGGGGGENLYFQG (i.e., a 10-glycine spacer flanked by two TEV protease motifs), followed in turn by residues 1–107 of the mAb light chain. DNA encoding SUMO fusion protein followed by a TEV protease motif was further included at the N terminus of that encoding the scFv in order to boost yield in Sf21 cells^40^. The pgpHFT-SUMO-scFv was then co-transfected with FlashBAC (Oxford Expression Technologies) into Sf21 insect cells as per the supplier’s manual. The seed virus was amplified to obtain high-titre viral stocks, which were then used to infect Sf21 cells grown in insect-XPRESS medium (Lonza). The protein product was purified from Sf21 cell culture supernatant by Q-Sepharose (GE Healthcare Lifesciences) anion-exchange chromatography (buffer A: 10 mM Tris, pH 8.0; buffer B: buffer A + 1.0 M NaCl) and digested with in-house-produced TEV protease to remove the SUMO domain and the single-chain linker domain. The desired Fv 24–60 product was then purified from the TEV-digested sample by MonoQ (GE Healthcare Lifesciences) anion-exchange chromatography (buffer A: 10 mM Tris, pH 8.0; buffer B: buffer A + 1.0 M NaCl) followed by Superdex 75 SEC in 20 mM Tris pH 8.0, 160 mM NaCl.

### Isothermal titration calorimetry

ITC experiments were performed using a MicroCal iTC200 instrument (Malvern Instruments) with the cell held at 25 °C and analysis undertaken with the manufacturer’s software within Origin (version 7.0; OriginLab).

For the titration of IGF-I against IGF-1RΔβ (*n* = 4 technical replicates) and of IGF-I against IGF-1RΔβ + Fv 24–60 (*n* = 2 technical replicates), IGF-I (GroPep Bioreagents; Australia) was prepared at concentrations of 80–100 μM (depending on the replicate) in TBSA. IGF-1RΔβ and IGF-1Δβ + Fv 24–60 prepared separately in TBSA at concentrations of 7–14 μM (depending on the replicate). The total number of injections in each titration was 16 at 180 s intervals, with the first injection being 1 μl and subsequent injections being 2.54 μl.

IGF-I CII was synthesized recombinantly as previously described^36^. Briefly, IGF-I CII was expressed in *Escherichia coli* in inclusion bodies, which were washed and solubilized in 8 M urea containing 40 mM glycine, 0.1 M Tris and 16 mM dithiothreitol (pH 2.0). Inclusion bodies were desalted on a Superdex 75 column (GE Healthcare Lifesciences) using the same buffer but with 1.6 mM dithiothreitol. Fractions containing the IGF-II fusion protein were pooled prior to folding in 2.5 M urea, 12.5 mM glycine, 0.7 M Tris, 5 mM EDTA, 0.5 mM dithiothreitol, 1 mM 2-hydroxyethyl disulphide, pH 9.1, and dilution to less than 0.1 mg mL^−1^. The fusion partner was removed by hydroxylamine cleavage (2 M urea, 1 M hydroxylamine, 0.1 M Tris pH 8.65, 37 °C, 22 h) and a final reverse-phase high-performance liquid chromatography clean-up. Purified protein was analysed by mass spectroscopy and N-terminal sequencing and was shown to have the correct mass and to be >95% pure. For ITC, IGF-I CII was prepared at concentrations of 50–65 μM (depending on the replicate) in TBSA and the solution then injected into separate volumes of IGF-1RΔβ + Fv 24–60 prepared at a concentration of 4.5 μM in TBSA (*n* = 2). The total number of injections in each titration was 11 at 180 s intervals, with the first injection being 1 μl and subsequent injections being 3.75 μl.

In all of the above titrations, technical replicates of individual experiments that employed identical concentrations were performed using the same sample. Errors for reported mean *K*_d_ values are the standard errors of the mean.

### Crystallization and data collection

The IGF-1RΔβ + Fv 24–60 complex was prepared by incubating IGF-1RΔβ with an excess of Fv 24–60 followed by SEC. Fractions containing the desired product were then concentrated to ~8 mg mL^−1^ in 10 mM HEPES, pH 7.5. Initial sparse-matrix crystallization screening was conducted in 96-well sitting-drop vapour diffusion format at the Collaborative Crystallization Centre (CSIRO, Parkville, Australia). The crystallization conditions were then refined in-house to 1.2 M (NH_4_)_2_SO_4_+0.1 M imidazole-malate, pH 7.0. For diffraction data collection, crystals were transferred to a cryo-protectant solution comprised of 1.2 M (NH_4_)_2_SO_4_+0.1 M imidazole-malate + 32% sucrose and then flash-frozen by cryo-plunging directly into a liquid nitrogen bath. All X-ray diffraction data were collected on the Australian Synchrotron beamline MX2^41^ at 100 K (*λ* = 0.9537 Å). Crystals containing IGF-1RΔβ + Fv 24–60 + IGF-I were obtained by soaking crystals of IGF-1RΔβ + Fv 24–60 in a solution identical to that of the mother liquor of crystallization but supplemented with increasing concentrations of IGF-I (up to ~1 mg mL^−1^). Cryo-protection and X-ray data collection for these crystals proceeded as above. Diffraction data for both crystal forms were integrated and scaled using the XDS package^42^; statistics are presented in Table 1. The resolution limit was set at being the maximum at which the *CC*_1/2_ statistic^43^ remained significant at the *P* = 0.001 level of significance. For molecular replacement, the diffraction data sets were further subjected to ellipsoidal truncation and anisotropic scaling using the Diffraction Anisotropy Server^44,45^.

### Structure determination and refinement

Molecular replacement (MR) structure solution of the apo form of IGF-1RΔβ in complex with Fv 24–60 was undertaken with PHASER^46^. Search objects for the L1-CR module and the L2 domain were obtained directly from the structure of the IGF1R L1-CR-L2 fragment (PDB entry 1IGR^25^); search objects for IGF-1RΔβ FnIII-1 domain and the (FnIII-2)-(FnIII-3) module were generated from their counterparts in the IRΔβ ectodomain structure (PDB entry 3LOH); whereas a search object for Fv 24–60 was generated from the structure of an anti-BclA scFv (PDB entry 3UMT; unpublished), modified by replacing all non-identical residues with serine using the FFAS03 server^47,48^. The MR search employed the anisotropy-corrected diffraction data set, as attempts using the complete data set failed. The initial model was refined against all data to 3.0 Å resolution using PHENIX^49^ iterated with manual rebuilding using COOT^50^. *N*-linked glycan residues were included where evident at Asn-X-Thr/Ser sequons. Refinement included translation / libration / screw (TLS) parameters, using TLS groups assigned by PHENIX. Within the refinement, the relative weighting of the X-ray and stereochemistry terms and of the X-ray and atomic displacement parameter terms were determined using the “automatic” protocol with PHENIX, rather than the program default option. Ramachandran plot percentages are favoured 92.3, allowed 6.6, outliers 1.1, rotamer outlier percentage is 0.2 and MolProbity^51^ all-atom clash score is 6.6. Final refinement statistics for both structures are in Table 1. Figures here and elsewhere were generated using Chimera^52^.

Structure solution for the IGF-I-bound crystals of IGF-1RΔβ + Fv 24-60 employed PHASER (again employing an anisotropy-corrected data set), searching with individual domains from the already refined Fv-complexed apo IGF-1RΔβ structure. Electron density for the IGF-I ligand was readily visible, bound to the L1 domain and αCT helix in a fashion effectively identical to that seen in its complex with the human insulin receptor domain L1 plus IGF-1R αCT (PDB entry 4XSS^27^), allowing ready model building and structure refinement within PHENIX and COOT as above, using all data to 3.26 Å resolution. *N*-linked glycan residues were included where evident at Asn-X-Thr/Ser sequons. Ramachandran plot percentages are favoured 92.2, allowed 7.3, outliers 0.5, rotamer outlier percentage is 1.4 and MolProbity^51^ all-atom clash score is 6.3. Final refinement statistics are in Table 1.

### Comparison of IGF-1R and IR quaternary structure

The relative dispositions of domains within IGF-1RΔβ compared to those of their counterparts in IRΔβ were computed using ProSMART^53^, using a fragment length of 15 residues.

### Mutant IGF-1R activation assays

Synthetic double-stranded complementary DNA (cDNA) fragments (Supplementary Table 3) incorporating either His774Ala, Ser788Ala, Asn789Ala, Phe790Ala, Phe792Ala or Phe790Ala/Phe792Ala (Integrated DNA Technologies) were cloned into an existing pcDNA3.1 plasmid incorporating the entire IGF-1R cDNA. Sequence-verified plasmids were transiently transfected into R^−^ cells (fibroblasts derived from IGF-1R knockout mice^54^, a gift from Dr Renato Baserga) and after 48 h cells expressing the IGF-1R mutants were stimulated with 100 nM IGF-I for 10 min. Cells were lysed^36^ in 20 mM HEPES, 150 mM NaCl, 1.5 mM MgCl_2_, 10% glycerol, 1% Triton X-100, 1 mM EGTA, pH 7.5 (lysis buffer) containing cOmplete protease inhibitor cocktail and PhosSTOP (Sigma-Aldrich), 2 mM Na_3_VO_4_, 100 mM NaF for 1 h at 4 °C; lysates were stored at −80 °C. Activation of the IGF-1R mutants was assessed by immunoblotting lysates with an antibody (#44–806G, Invitrogen; 1:2000 dilution, raised in rabbit) specific for phosphorylated Tyr1158/Tyr1162/Tyr1163 (in the IGF-1R kinase activation loop). Total receptor expression was measured by probing with an IGF-1R antibody (3027S, Cell Signaling; 1:1000 dilution, raised in rabbit) and an anti-tubulin antibody (Invitrogen; 1:1000 dilution, raised in mouse) was used as a loading control. Anti-rabbit IR dye 680RD and anti-mouse IR dye 800CW (Licor; 1:50,000 dilution) were used as secondary antibodies. Quantitation of the blots was achieved using the Image Studio Lite quantitation software (LI-COR Biosciences) as follows. First, the IGF-1R expression levels were normalized to the tubulin expression in each lane, as were the levels of Tyr1158/Tyr1162/Tyr1163 phosphorylation. The fold activation above basal was then calculated by comparing levels of Tyr1158/Tyr1162/Tyr1163 phosphorylation in non-stimulated and stimulated samples. Data represent *n* = 3 technical replicates and were analysed using one-way analysis of variance within Prism 7.0 (GraphPad).

### Mutant IGF-1R affinity assays

After 72 h of transfection of WT and mutant IGF-IR into R^−^ cells, cells were lysed with lysis buffer (20 mM HEPES, 150 mM NaCl, 1.5 mM MgCl_2_, 10% v/v glycerol, 1% v/v Triton X-100, 1 mM EGTA (pH 7.5)) for 1 h at 4 °C. Lysates were centrifuged for 10 min at 3500 rpm and then 100 μl was added per well to a white Greiner Lumitrac 600 plate previously coated with anti-IGF-1R antibody 24–31^23^ and blocked with 0.5% bovine serum albumin in TBST (20 mM Tris, 150 mM NaCl, 0.05% v/v Tween-20). P6 cells (a gift from Dr Renato Baserga) were used as a positive control for over-expression of the wild-type IGF-IR^55^. Plates were incubated at 4 °C for 16 h.

Europium-labelled receptor-grade human IGF-I (Gropep Bioreagents; Australia) was prepared as instructed by the manufacturer (DELFIA Eu-labelling kit, Perkin Elmer). Approximately 3 × 10^6^ fluorescent counts of europium-labelled IGF-I were added to each well along with IGF-I competitor (0–300 nM) in triplicate and incubated for 16 h at 4 °C. Wells were washed three times with TBST, followed by addition of DELFIA enhancement solution (100 μl per well). After 10 min, time-resolved fluorescence was measured using 340 nm excitation and 612 nm emission filters with a Victor X4 2030 Multilabel Reader (Perkin Elmer). IC_50_ values were calculated using Prism 7.0 (GraphPad) for curve-fitting of a one-site competition model.

### Modelling negative cooperativity of IGF-1R and IR

The experimental data for negative cooperativity of IGF-1R and IR were those described previously^18^ and are provided for completeness here in Supplementary Table 4 by permission of Professor Pierre De Meyts. As indicated in the Discussion, the initial binding of ligand to the “closed” form of receptor can be explained by an induced fit model or by transient receptor opening, the latter effectively being described by the HO model^18^ upon reversal of the percentage of times that the receptor spends in its respective open and closed conformations. The exact nature of this binding event is not important for the modelling presented here, as its sequential components can be grouped into a single reaction (Supplementary Figure 5a) that represents high-affinity receptor binding (and receptor activation). Binding of a second ligand would then lead to either an asymmetric or symmetric receptor conformation. Both cases need to be considered. (i) In case of an asymmetric conformation, the second ligand hypothetically binds to a partially open site 1 of the alternate pair of binding sites, without it engaging site 2 (Supplementary Figure 5b). This interaction is expected to have a lower affinity to that of an interaction engaging both sites. In order for the negative cooperativity to occur, the asymmetric conformation is presumed to transition between the two possible states in which the ligand initially bound with high affinity disengages site 2 (leading to low affinity) and the ligand initially bound with low affinity to site 1 alone engages both sites (leading to high affinity) (Supplementary Figure 5b). This mechanism is formally identical to negative cooperativity within HO model, and thus the HO formalism can be applied (albeit with an alternative structural interpretation). (ii) In the case of a symmetric conformation (Supplementary Figure 5c), the two sites have ligand bound with identical affinity. This affinity is expected to be reduced compared to that of the singly bound receptor, as otherwise we would have a receptor with two high-affinity sites, contradicting binding data that demonstrate that there is only one high-affinity site per holo-receptor^18,38,56^, unlike the soluble IR ectodomain that has two equal lower-affinity sites^57^. Indeed, it is plausible that symmetrical opening of the receptor domains to accommodate two ligands requires distortion of the receptor structure in energetically costly fashion that reduces ligand affinity. The binding of a third insulin molecule is proposed to account for the ascending phase of accelerated dissociation for IR^38^. IGF-1R lacks this part of the curve and thus, for simplicity, binding of the third ligand will be considered only in the case of insulin binding to IR. Additional separation (“opening”) of the receptor domains may be required to accommodate the third ligand (Supplementary Figure 5d), presumably via an energetically unfavourable process that results in very low affinity for that ligand. It is proposed that binding of the third ligand “locks” the tracer in the bound state in the experiment for accelerated dissociation, and tracer dissociation can only occur after the cold ligand dissociates^38^. Taking into account the above described binding reactions, the model proposed here with the use of doubly liganded, symmetrical receptor conformation leads to a compact binding scheme of the ligand–receptor interaction (Supplementary Figure 5e). It should be noted that this binding scheme is applicable only to the experimental conditions described above. For example, receptor intermediaries with two or three hot ligand molecules bound were excluded from the reaction scheme, since they would not be formed in any significant quantities at 10 pM ligand concentration. Similarly, intermediates with only cold ligand molecules bound were eliminated due to tracer pre-binding. Endocytosis is, however, included, as even though the binding data were derived from experiments performed at 16 °C, endocytosis at this temperature cannot be totally excluded^18^. Thus, as within the HO model, it is assumed that upon activation of inactive receptor intermediary, R_000_, the active intermediaries such as R_h00,_ R_0c0_, R_hc0_ or R_hcc_ (see Supplementary Figure 5e) are internalized with an internalization rate constant *k*_end_. Upon internalization, it is assumed that ligand dissociates instantly which leads to accumulation of hot ligand, Lig_end_, and internalized receptor, R_cyt_, inside the cells. The internalized receptor, R_cyt_, is recycled back to the plasma membrane with an exocytosis rate constant, *k*_ex_. The internalized ligand, Lig_end_, is recycled out of cells (either intact or degraded) with an exocytosis rate constant, *k*_ex_. The binding of two species of ligand (hot and cold) in the presence of endo- and exocytosis and under conditions of no ligand depletion can be described by a system of ordinary differential equations shown in Supplementary Figure 6. The rate constants for endocytosis and exocytosis in IM9 cells were taken from the HO model^18^. The initial values for *a*_1_ and *d*_1_ (high affinity) site, *a*_2_ and *d*_2_ (low affinity symmetrical conformation) and *a*_3_ and *d*_3_ (describing binding of the third insulin molecule) were also taken from the HO model^18^ and manually optimized to achieve a fit to experimental data for accelerated dissociation at 20 min while keeping the high-affinity site constrained to *K*_d_ = 0.12 nM for IGF-I and *K*_d_ = 0.2 nM for insulin and the low-affinity site to *K*_d_ = 4.3 nM in case of IGF-I and *K*_d_ = 6 nM in case of insulin. Simulations were performed using Mathematica v11.0 (Wolfram). The optimized parameter values are shown in Supplementary Table 5. No attempt was made to obtain a best fit of parameters or to establish if the identified parameter set is unique; nevertheless, the identified set of parameters leads to good agreement with experimental data (Fig. 8b).

### Data availability

The coordinates of the structures determined here and their associated structure factors have been deposited in the Protein Data Bank (accession codes 5U8R and 5U8Q). Other data are available from the corresponding author upon reasonable request.

## Electronic supplementary material

Supplementary Information

## References

[1] Ullrich A (1986). Insulin-like growth factor I receptor primary structure: comparison with insulin receptor suggests structural determinants that define functional specificity. EMBO J..

[2] Pollak MN, Schernhammer ES, Hankinson SE (2004). Insulin-like growth factors and neoplasia. Nat. Rev. Cancer.

[3] Pollak M (2012). The insulin receptor/insulin-like growth factor receptor family as a therapeutic target in oncology. Clin. Cancer Res..

[4] Adams TE, Epa VC, Garrett TP, Ward CW (2000). Structure and function of the type 1 insulin-like growth factor receptor. Cell. Mol. Life Sci..

[5] Forbes BE, McCarthy P, Norton RS (2012). Insulin-like growth factor binding proteins: a structural perspective. Front. Endocrinol. (Lausanne).

[6] Brown J (2007). Structure and functional analysis of the IGF-II/IGF2R interaction. EMBO J..

[7] McKern NM (2006). Structure of the insulin receptor ectodomain reveals a folded-over conformation. Nature.

[8] Croll TI (2016). Higher-resolution structure of the human insulin receptor ectodomain: multi-modal inclusion of the insert domain. Structure.

[9] Smith BJ (2010). Structural resolution of a tandem hormone-binding element in the insulin receptor and its implications for design of peptide agonists. Proc. Natl. Acad. Sci. USA.

[10] Menting JG (2013). How insulin engages its primary binding site on the insulin receptor. Nature.

[11] Menting JG (2014). Protective hinge in insulin opens to enable its receptor engagement. Proc. Natl. Acad. Sci. USA.

[12] Kristensen C, Andersen AS, Østergaard S, Hansen PH, Brandt J (2002). Functional reconstitution of insulin receptor binding site from non-binding receptor fragments. J. Biol. Chem..

[13] Brzozowski AM (2002). Structural origins of the functional divergence of human insulin-like growth factor-I and insulin. Biochemistry.

[14] De Meyts P (2015). Insulin/receptor binding: the last piece of the puzzle?: What recent progress on the structure of the insulin/ receptor complex tells us (or not) about negative cooperativity and activation. Bioessays.

[15] Gauguin L (2008). Alanine scanning of a putative receptor binding surface of insulin-like growth factor-I. J. Biol. Chem..

[16] Alvino CL (2009). A novel approach to identify two distinct receptor binding surfaces of insulin-like growth factor II. J. Biol. Chem..

[17] De Meyts P, Whittaker J (2002). Structural biology of insulin and IGF1 receptors: implications for drug design. Nat. Rev. Drug. Discov..

[18] Kiselyov VV, Versteyhe S, Gauguin L, De Meyts P (2009). Harmonic oscillator model of the insulin and IGF1 receptors’ allosteric binding and activation. Molec. Sys. Biol..

[19] Kavran JM (2014). How IGF-1 activates its receptor. Elife.

[20] Lee J, Miyazaki M, Romeo GR, Shoelson SE (2014). Insulin receptor activation with transmembrane domain ligands. J. Biol. Chem..

[21] Whitten AE (2009). Solution structure of ectodomains of the insulin receptor family: the ectodomain of the type 1 insulin-like growth factor receptor displays asymmetry of ligand binding accompanied by limited conformational change. J. Mol. Biol..

[22] Surinya KH (2008). An investigation of the ligand binding properties and negative cooperativity of soluble insulin-like growth factor receptors. J. Biol. Chem..

[23] Soos MA (1992). A panel of monoclonal antibodies for the type I insulin-like growth factor receptor. Epitope mapping, effects on ligand binding, and biological activity. J. Biol. Chem..

[24] Koide S (2009). Engineering of recombinant crystallization chaperones. Curr. Opin. Struct. Biol..

[25] Garrett TP (1998). Crystal structure of the first three domains of the type-1 insulin-like growth factor receptor. Nature.

[26] Sparrow LG (2008). N-linked glycans of the human insulin receptor and their distribution over the crystal structure. Protein Struct. Funct. Bioinform..

[27] Menting JG (2015). Structural congruency of ligand binding to the insulin and insulin/type 1 insulin-like growth factor hybrid receptors. Structure.

[28] Lawrence MC, Colman PM (1993). Shape complementarity at protein/protein interfaces. J. Mol. Biol..

[29] Houde D, Demarest SJ (2011). Fine details of IGF-1R activation, inhibition, and asymmetry determined by associated hydrogen/deuterium-exchange and peptide mass mapping. Structure.

[30] Lou M (2006). The first three domains of the insulin receptor differ structurally from the insulin-like growth factor 1 receptor in the regions governing ligand specificity. Proc. Natl. Acad. Sci. USA.

[31] Denley A (2005). Structural and functional characteristics of the Val44Met insulin-like growth factor I missense mutation: correlation with effects on growth and development. Mol. Endocrinol..

[32] Kristensen C (1995). A single-chain insulin-like growth factor I/insulin hybrid binds with high affinity to the insulin receptor. Biochem. J..

[33] Nanjo K (1986). Diabetes due to secretion of a structurally abnormal insulin (insulin Wakayama). Clinical and functional characteristics of [LeuA3] insulin. J. Clin. Invest..

[34] Hoyne PA, Elleman TC, Adams TE, Richards KM, Ward CW (2000). Properties of an insulin receptor with an IGF-1 receptor loop exchange in the cysteine-rich region. FEBS Lett..

[35] Keyhanfar M, Booker GW, Whittaker J, Wallace JC, Forbes BE (2007). Precise mapping of an IGF-I-binding site on the IGF-1R. Biochem. J..

[36] Denley A (2004). Structural determinants for high-affinity binding of insulin-like growth factor II to insulin receptor (IR)-A, the exon 11 minus isoform of the IR. Mol. Endocrinol..

[37] Denley A, Cosgrove LJ, Booker GW, Wallace JC, Forbes BE (2005). Molecular interactions of the IGF system. Cytokine Growth Factor Rev..

[38] De Meyts P (1994). The structural basis of insulin and insulin-like growth factor-I receptor binding and negative co-operativity, and its relevance to mitogenic versus metabolic signalling. Diabetologia.

[39] Xu Y (2010). Crystal structure of the entire ectodomain ofgp130: insights into the molecular assembly of the tall cytokine receptor complexes. J. Biol. Chem..

[40] Liu L, Spurrier J, Butt TR, Strickler JE (2008). Enhanced protein expression in the baculovirus/insect cell system using engineered SUMO fusions. Protein Expr. Purif..

[41] McPhillips TM (2002). Blu-Ice and the Distributed Control System: software for data acquisition and instrument control at macromolecular crystallography beamlines. J. Synchrotron Radiat..

[42] Kabsch W (2010). Integration, scaling, space-group assignment and post-refinement. Acta Crystallogr. D Biol. Crystallogr..

[43] Karplus PA, Diederichs K (2012). Linking crystallographic model and data quality. Science.

[44] UCLA-DOE LAB — Diffraction Anisotropy Server, http://services.mbi.ucla.edu/anisoscale/ (2012).

[45] Strong M (2006). Toward the structural genomics of complexes: crystal structure of a PE/PPE protein complex from *Mycobacterium tuberculosis*. Proc. Natl. Acad. Sci. USA.

[46] McCoy AJ (2007). Phaser crystallographic software. J. Appl. Crystallogr..

[47] Jaroszewski L, Rychlewski L, Li Z, Li W, Godzik A (2005). FFAS03: a server for profile--profile sequence alignments. Nucleic Acids Res..

[48] Fold & Function Assignment System, http://ffas.sanfordburnham.org/ (2011).

[49] Adams PD (2010). PHENIX: a comprehensive Python-based system for macromolecular structure solution. Acta Crystallogr. D Biol. Crystallogr.

[50] Emsley P, Cowtan K (2004). Coot: model-building tools for molecular graphics. Acta Crystallogr. D Biol. Crystallogr..

[51] Davis IW (2007). MolProbity: all-atom contacts and structure validation for proteins and nucleic acids. Nucleic Acids Res..

[52] Pettersen EF (2004). UCSF Chimera—a visualization system for exploratory research and analysis. J. Comp. Chem..

[53] Nicholls RA, Fischer M, McNicholas S, Murshudov GN (2014). Conformation-independent structural comparison of macromolecules with ProSMART. Acta Crystallogr. D Biol. Crystallogr..

[54] Sell C (1994). Effect of a null mutation of the insulin-like growth factor I receptor gene on growth and transformation of mouse embryo fibroblasts. Mol. Cell. Biol..

[55] Pietrzkowski Z (1992). Constitutive expression of insulin-like growth factor 1 and insulin-like growth factor 1 receptor abrogates all requirements for exogenous growth factors. Cell. Growth Differ..

[56] Schäffer L (1994). A model for insulin binding to the insulin receptor. Eur. J. Biochem..

[57] Markussen J, Halstrøm J, Wiberg FC, Schäffer L (1991). Immobilized insulin for high capacity affinity chromatography of insulin receptors. J. Biol. Chem..

