# Supplementary material for: How ligand binds to the type 1 insulin-like growth factor receptor
Source: Nat Commun. 2018 Feb 26;9:821. doi: 10.1038/s41467-018-03219-7 (PMC5826941; doi:10.1038/s41467-018-03219-7)
Supplement: Supplementary file 1 — Supplementary Information [file 41467_2018_3219_MOESM1_ESM.pdf]

SUPPLEMENTARY INFORMATION

How ligand binds to the Type 1 insulin-like growth factor receptor

Xu *et al.*

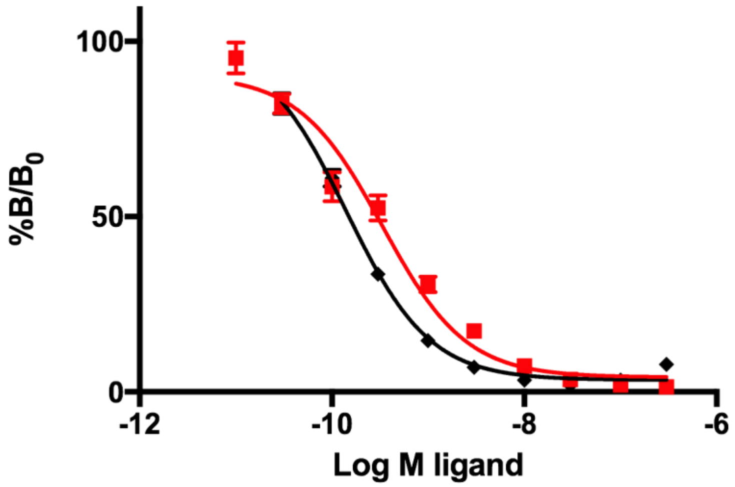

Supplementary Figure 1. Affinity of IGF-1RΔβ for IGF-I and IGF-II.

Competition ligand-binding assay (IGF-I black, IGF-II red) performed with IGF-1RΔβ plate-captured *via* mAb 24-31[1], with Eu^{2+} labelled ligand competed with unlabelled ligand. Error bars reflect standard deviation of the mean of n = 9 technical replicates for IGF-I and n = 6 technical replicates for IGF-II (error bars appear absent when smaller than the marker size). The IC_{50} values of IGF-I and IGF-II for IGF-1RΔβ are 0.14 nM and 0.33 nM, respectively, (with the 95% confidence intervals being 0.12-0.17 nM and 0.23-0.48 nM, respectively).

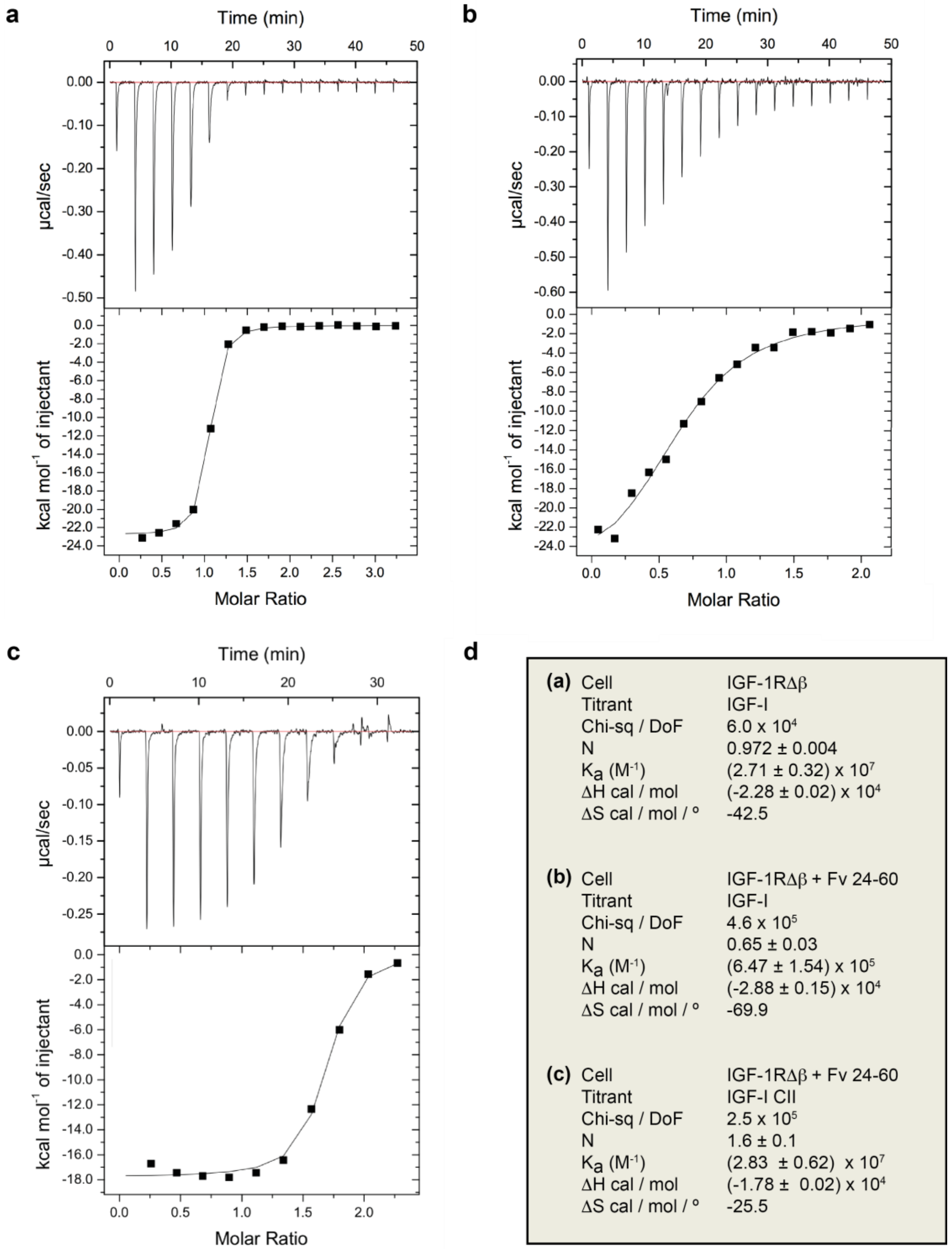

Supplementary Figure 2. Isothermal titration calorimetry of ligand binding to IGF-1RΔβ

(**a**) Representative ITC thermogram for IGF-I titrated against IGF-1RΔβ; (**b**) Representative ITC thermogram for IGF-I titrated against IGF-1RΔβ + Fv 24-60, and (**c**) Representative ITC thermogram for IGF-I CII titrated against IGF-1RΔβ + Fv 24-60. (**d**) Thermodynamic parameters for the single experiments (a), (b) and (c) as calculated by the ITC manufacturer's software using a single-site model (one ligand per receptor monomer). Full details are provided in the Methods section (*q.v.*).

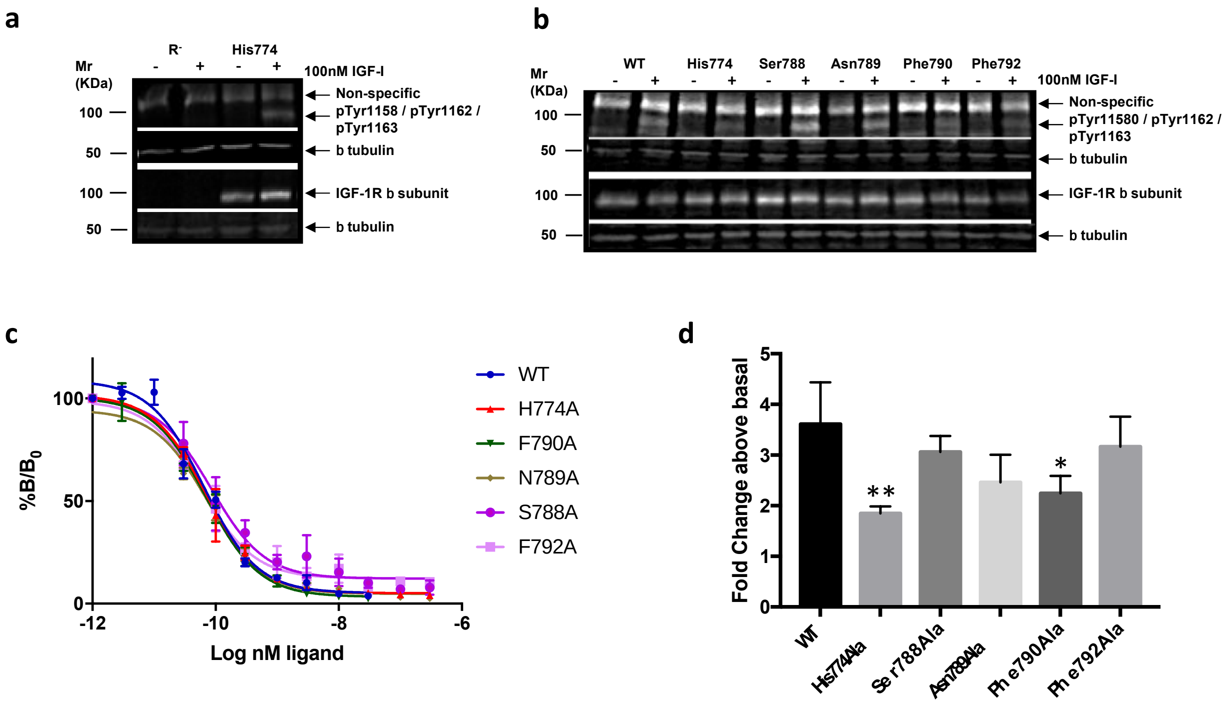

Supplementary Figure 3. Wild-type and mutant IGF-1R: relative affinity for and relative activation by IGF-I.

(**a**) Western blot showing degree of IGF-1R expression in untransfected R⁻ fibroblasts compared to that of His774Ala mutant IGF-1R upon transfection into R⁻ fibroblasts, as well as the degree of activation by IGF-I. IGF-1R is seen to not be expressed in the R⁻ fibroblasts. (**b**) Western blot showing degree of expression of wild-type and mutant IGF-1R upon transfection into R⁻ fibroblasts and degree of activation by IGF-I. For uncropped images of the gels in (a) and (b), see **Supplementary Figure 4**. (**c**) Competition binding experiments to determine IGF-I binding affinities of IGF-1R alanine mutants. Immuno-captured solubilized wild-type (WT) or mutant IGF-1R were incubated with europium-labelled IGF-I with or without increasing concentrations of IGF-I. Results are expressed as a percentage of binding in the absence of competing ligand (%B/B_0) and the data points are the mean ± s.e.m. of *n* = 3 technical replicates. Error bars are shown when greater than the size of the symbols. The assay was conducted once. Determined affinities are presented in **Supplementary Table 2** (*q.v.*) and are not significantly different to that of WT at the 95% degree of confidence. (**d**) Relative degree of activation of mutant receptors with respect to wild-type. Data are from two experiments and *n* = 3 technical replicates and were analysed using one-way ANOVA. *: P = 0.0022, **: P =0.0192, not significant: P > 0.05. Full details are provided in the Methods section (*q.v.*).

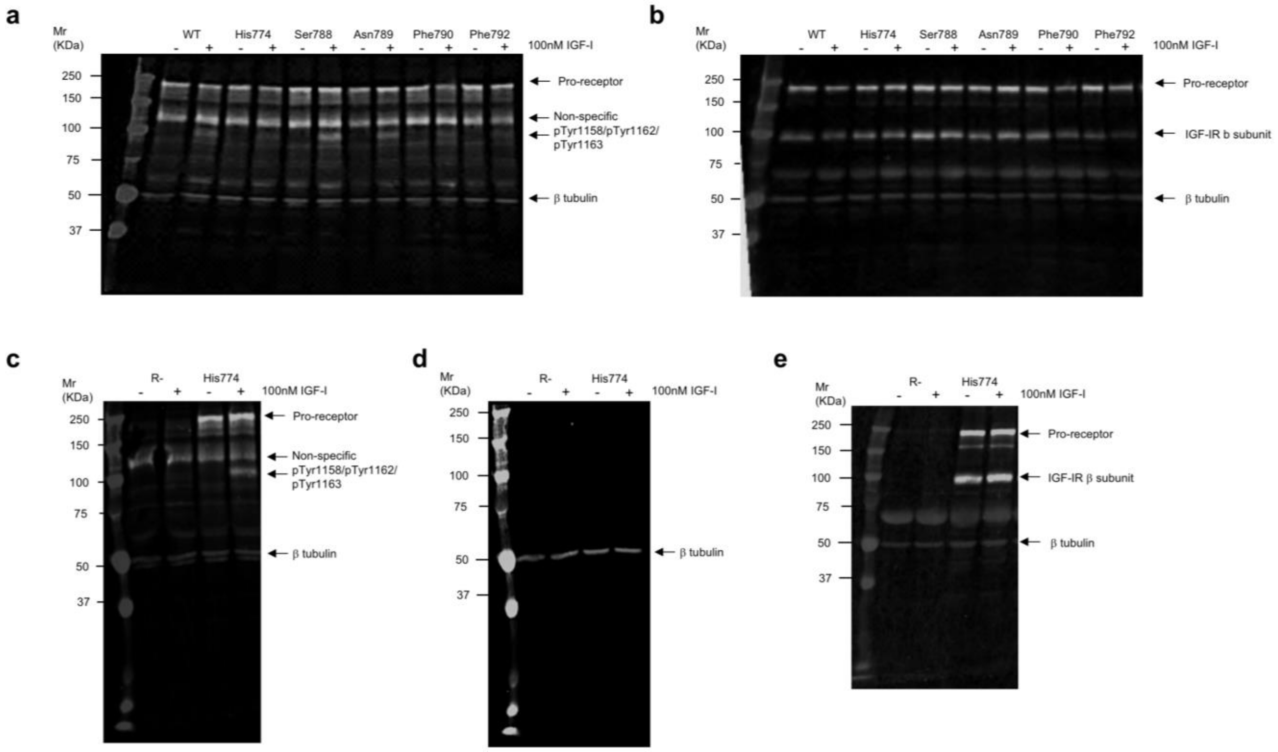

Supplementary Figure 4. **Uncropped images of the respective gels used to create the montages presented in Supplementary Figures 3a and 3b.** (**a**) Uncropped image of the gel from which the upper two components of the montage in Supplementary Figure 3a were derived. (**b**) Uncropped image of the gel from which the lower two components of the montage in Supplementary Figure 3a were derived. (**c**) Uncropped image of the gel from which the top component of the montage in Supplementary Figure 3b was derived. (**d**) Uncropped image of the gel from which the tubulin control within the montage in Supplementary Figure 3b was derived. (**e**) Uncropped image of the gel from which the lower two components of the montage in Supplementary Figure 3b were derived.

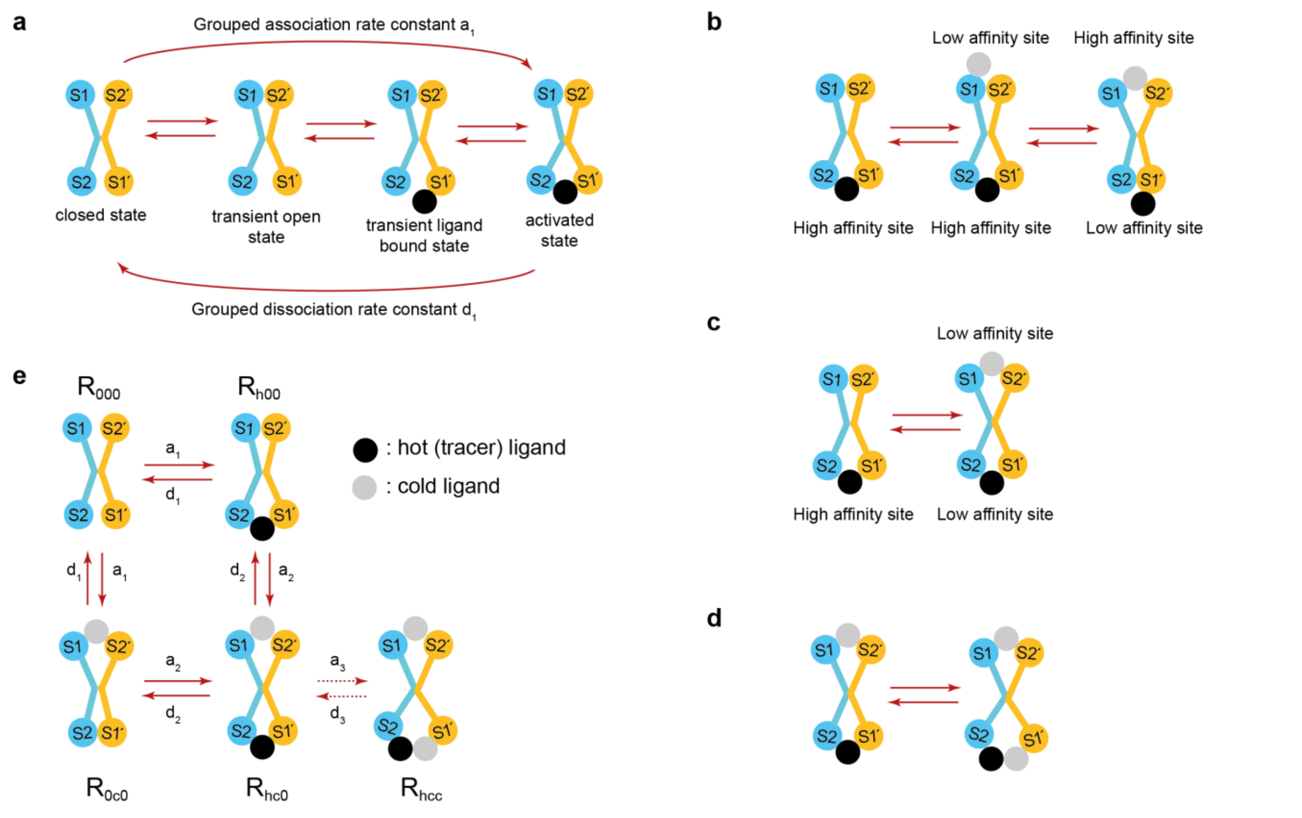

Supplementary Figure 5. Reaction schemes for negative cooperativity of ligand binding to IGF-1R and IR.

(**a**) Binding of the first ligand molecule to receptor. (**b**) Binding of the second ligand molecule with formation of a putative non-symmetric receptor conformation. (**c**) Binding of the second ligand molecule with formation of a doubly-liganded, symmetric receptor conformation. (**d**) Binding of the third ligand molecule. (**e**) Reaction scheme for binding of two ligand species (hot and cold). a_1 and d_1: association and dissociation rate constants for the high-affinity site. a_2 and d_2: association and dissociation rate constants for doubly-liganded, symmetrical receptor conformation. a_3 and d_3: association and dissociation rate constant for binding of a third insulin molecule to IR (not applicable to IGFs binding to IGF-1R). Nomenclature in all Panels is as follows:- S1, S2: site 1 and site 2 on one receptor monomer; S1′, S2′: site 1 and site 2 on the opposing receptor monomer. Black filled circle: hot ligand (*i.e.*, IGF-I, IGF-II or insulin). Open circle: cold ligand (*i.e.*, IGF-I, IGF-II or insulin).

$$R_{000}'(t) = -a_1 \times R_{000}(t) \times \mathrm{Lig}_{hot} + d_1 \times R_{h00}(t) - a_1 \times R_{000}(t) \times \mathrm{Lig}_{cold} + d_1 \times R_{0c0}(t) + k_{ex} \times R_{cyt}(t)$$

$$R_{h00}'(t) = a_1 \times R_{000}(t) \times \mathrm{Lig}_{hot} - d_1 \times R_{h00}(t) - a_2 \times R_{h00}(t) \times \mathrm{Lig}_{cold} + d_2 \times R_{hc0}(t) - k_{end} \times R_{h00}(t)$$

$$R_{hc0}'(t) = a_2 \times R_{h00}(t) \times \mathrm{Lig}_{cold} - d_2 \times R_{hc0}(t) + a_2 \times R_{0c0}(t) \times \mathrm{Lig}_{hot} - d_2 \times R_{hc0}(t) -$$
$$a_3 \times R_{hc0}(t) \times \mathrm{Lig}_{cold} + d_3 \times R_{hcc}(t) - k_{end} \times R_{hc0}(t)$$

$$R_{hcc}'(t) = a_3 \times R_{hc0}(t) \times \mathrm{Lig}_{cold} - d_3 \times R_{hcc}(t) - k_{end} \times R_{hcc}(t)$$

$$R_{0c0}'(t) = a_1 \times R_{000}(t) \times \mathrm{Lig}_{cold} - d_1 \times R_{0c0}(t) - a_2 \times R_{0c0}(t) \times \mathrm{Lig}_{hot} + d_2 \times R_{hc0}(t) - k_{end} \times R_{0c0}(t)$$

$$R_{cyt}'(t) = -k_{ex} \times R_{cyt}(t) + k_{end} \times R_{h00}(t) + k_{end} \times R_{hc0}(t) + k_{end} \times R_{hcc}(t) + k_{end} \times R_{0c0}(t)$$

$$\mathrm{Lig}_{end}'(t) = -k_{ex} \times \mathrm{Lig}_{end}(t) + k_{end} \times R_{h00}(t) + k_{end} \times R_{hc0}(t) + k_{end} \times R_{hcc}(t)$$

Supplementary Figure 6. System of differential equations describing negative cooperativity of ligand binding to IR and IGF-1R.

Lig_{hot} is the concentration of monomeric hot ligand in case of insulin or a total ligand concentration in case of IGF-I. Lig_{cold} is the concentration of monomeric cold ligand in case of insulin or a total concentration in case of IGF-I. The concentration of monomeric insulin is calculated by subtracting the concentration of dimeric insulin from the total concentration of insulin. Insulin is assumed to dimerize with $K_d = 7.14$ μM[2,3]. For IGF-I binding to IGF-1R, a_3 and d_3 are zero. Here, prime (') denotes differentiation with respect to time, *t*.

IGF-I residue	αCT contacts	L1 contacts	IGF-I mutant affinity for IGF-1R (% wt)	Reference
Gly7	Glu693, His697	-	-	-
Ala8	Glu693	Glu91	Leu 17%	4
Val11	His697, Phe701	Arg59	Thr 30%<br>Ile 160%<br>Ala 40%	5<br>5<br>6
Asp12	-	Arg59	Ala 40%	6
Leu14	Phe701	-	-	-
Gln15	-	Leu33, Arg59	Ser 80%<br>Ala 51%<br>Glu 60%	4<br>6<br>6
Gly22	-	Asn11	-	-
Phe23	Phe701	Arg10, Asn11, Leu33	Gly 2%	7
Tyr24	Val702, Pro703, Arg704	Arg10	Ser 6%<br>Leu 3%	8<br>8
Phe25	Ile700	Asp8, Arg10	-	-
Gln40	Phe695, Asn698, Ser699	-	-	-
Gly42	Asn698	-	-	-
Ile43	His697, Asn698	-	-	-
Val44	Asn694, Asn698	-	Met 1%	9
Glu58	Arg704	-	-	-
Met59	Pro703, Arg704	-	Phe 20%	4
Tyr60	Phe701, Pro703		Leu 5%<br>Phe 100%	10<br>5

Supplementary Table 1. Site 1 contacts of IGF-I with IGF-1RΔβ.

Listed are contacts identified in this study as well as in literature-derived data[11] for the altered affinity of single-site IGF-I mutation at the respective sites. Contacts were computed using the program CONTACT within the CCP4 suite[12] with default distance criteria.

	IGF-I binding affinity (IC_{50}, 10^{-11} M)	Affinity relative to WT
WT	6.4 (4.8-8.6)[1]	1
His774Ala	7.0 (5.5-8.9)	1.1
Phe790Ala	7.0 (5.5-9.0)	1.1
Asn789Ala	7.5 (5.5-9.0)	1.2
Ser788Ala	8.4 (5.8-9.9)	1.3
Phe792Ala	5.9 (5.2-14.6)	0.9

[1]Numbers in parentheses refer to the 95% confidence interval of the IC_{50} values based on a single-site fit to the data.

Supplementary Table 2. IGF-I binding affinities of IGF-1R and mutants

IGF-IR mutation	EcoRV/SacII cDNA fragment sequence (mutation underlined)
His774Ala	GATATCTAACCTGAGGCCCTTCACCCTGTACAGAATCGACATCGCCA<br>GCTGCAACCACGAGGCTGAGAAGCTGGGCTGCAGCGCCTCCAACTT<br>CGTGTTCGCCAGAACCATGCCCGCCGAGGGCGCCGATGACATCCCTG<br>GACCCGTGACATGGGAACCGCGG
Ser788Ala	GATATCTAACCTGAGGCCCTTCACCCTGTACAGAATCGACATCCACA<br>GCTGCAACCACGAGGCTGAGAAGCTGGGCTGCAGCGCCGCCAACTT<br>CGTGTTCGCCAGAACCATGCCCGCCGAGGGCGCCGATGACATCCCTG<br>GACCCGTGACATGGGAACCGCGG
Asn789Ala	GATATCTAACCTGAGGCCCTTCACCCTGTACAGAATCGACATCCACA<br>GCTGCAACCACGAGGCTGAGAAGCTGGGCTGCAGCGCCTCCGCCTT<br>CGTGTTCGCCAGAACCATGCCCGCCGAGGGCGCCGATGACATCCCTG<br>GACCCGTGACATGGGAACCGCGG
Phe790Ala	GATATCTAACCTGAGGCCCTTCACCCTGTACAGAATCGACATCCACA<br>GCTGCAACCACGAGGCTGAGAAGCTGGGCTGCAGCGCCTCCAACGC<br>CGTGTTCGCCAGAACCATGCCCGCCGAGGGCGCCGATGACATCCCTG<br>GACCCGTGACATGGGAACCGCGG
Phe792Ala	GATATCTAACCTGAGGCCCTTCACCCTGTACAGAATCGACATCCACA<br>GCTGCAACCACGAGGCTGAGAAGCTGGGCTGCAGCGCCTCCAACTT<br>CGTGGCCGCCAGAACCATGCCCGCCGAGGGCGCCGATGACATCCCT<br>GGACCCGTGACATGGGAACCGCGG
Phe790Ala/<br>Phe792Ala	GATATCTAACCTGAGGCCCTTCACCCTGTACAGAATCGACATCCACA<br>GCTGCAACCACGAGGCTGAGAAGCTGGGCTGCAGCGCCTCCAACGC<br>CGTGGCCGCCAGAACCATGCCCGCCGAGGGCGCCGATGACATCCCT<br>GGACCCGTGACATGGGAACCGCGG

Supplementary Table 3. Double-stranded cDNA fragments employed to create alanine mutation(s) within IGF-1R cDNA. These fragments (synthesized by Integrated DNA Technologies) were cloned (EcoRV/SacII) into an existing pcDNA3.1 plasmid incorporating the entire IGF-1R cDNA and modified to include a set of unique restriction sites distributed across the IGF-1R coding region.

Concentration cold IGF-I (nM)	<Bound/$Bound_0$>	s.e.m.
0.0132	0.91	0.10
0.0407	0.90	0.10
0.132	0.86	0.10
0.407	0.83	0.10
1.32	0.81	0.11
4.07	0.61	0.04
13.2	0.47	0.04
40.7	0.34	0.04
132	0.33	0.05
407	0.33	0.05

Concentration cold insulin (nM)	<Bound/$Bound_0$>	s.e.m.
0.0166	0.78	0.08
0.0513	0.83	0.08
0.166	0.78	0.08
0.513	0.71	0.07
1.66	0.53	0.06
5.13	0.37	0.05
16.6	0.25	0.04
51.3	0.21	0.04
166	0.22	0.04
513	0.26	0.05
1660	0.35	0.05
16600	0.62	0.08

Supplementary Table 4. Negative cooperativity data for IGF-1R (upper table) and IR (lower table). These data are those originally employed in ref. 3 and are provided here for completeness by permission of Prof. Pierre De Meyts. s.e.m. = standard deviation of the mean of n = 3 technical replicates.

Parameter	Description	IGF-1R	IR
a_1	Association rate constant for first ligand bound	0.000692 nM^{-1} s^{-1}	0.001078 nM^{-1} s^{-1}
d_1	Dissociation rate constant for first ligand bound	0.0000814 s^{-1}	0.0002066 s^{-1}
a_2	Association rate constant for second ligand bound	0.000346 nM^{-1} s^{-1}	0.000425 nM^{-1} s^{-1}
d_2	Dissociation rate constant for second ligand bound	0.0015 s^{-1}	0.00255 s^{-1}
a_3	Association rate constant for third ligand bound	n/a	0.000009765 nM^{-1} s^{-1}
d_3	Dissociation rate constant for third ligand bound	n/a	0.01 s^{-1}
k_{end}	Endocytosis rate constant	0.000055 s^{-1}	0.000033 s^{-1}
k_{ex}	Exocytosis rate constant	0.0000056 s^{-1}	0.000023 s^{-1}

Supplementary Table 5. Optimized parameter values for ligand binding to IGF-1R and IR within the symmetrical binding model (Fig. 8a).

Supplementary References

1. Soos, M.A. *et al.* A panel of monoclonal antibodies for the type I insulin-like growth factor receptor. Epitope mapping, effects on ligand binding, and biological activity. *J. Biol. Chem.* **267**, 12955-12963 (1992).
2. Pekar, A.H. & Frank, B.H. Conformation of proinsulin. A comparison of insulin and proinsulin self-association at neutral pH. *Biochemistry* **11**, 4013-4016 (1972).
3. Kiselyov, V.V., Versteyhe, S., Gauguin, L. & De Meyts, P. Harmonic oscillator model of the insulin and IGF1 receptors' allosteric binding and activation. *Molec. Sys. Biol.* **5**, 243 (2009).
4. Shooter, G.K. *et al.* Insulin-like growth factor (IGF)-I A- and B-domain analogues with altered type 1 IGF and insulin receptor binding specificities. *J. Mol. Endocrinol.* **17**, 237-246 (1996).
5. Hodgson, D.R., May, F.E. & Westley, B.R. Mutations at positions 11 and 60 of insulin-like growth factor 1 reveal differences between its interactions with the type I insulin-like-growth-factor receptor and the insulin receptor. *Eur. J. Biochem.* **233**, 299-309 (1995).
6. Jansson, M., Uhlen, M. & Nilsson, B. Structural changes in insulin-like growth factor (IGF) I mutant proteins affecting binding kinetic rates to IGF binding protein 1 and IGF-I receptor. *Biochemistry* **36**, 4108-4117 (1997).
7. Hodgson, D.R., May, F.E. & Westley, B.R. Involvement of phenylalanine 23 in the binding of IGF-1 to the insulin and type I IGF receptor. *Regul. Pept.* **66**, 191-196 (1996).
8. Cascieri, M.A. *et al.* Mutants of human insulin-like growth factor I with reduced affinity for the type 1 insulin-like growth factor receptor. *Biochemistry* **27**, 3229-3233 (1988).
9. Denley, A. *et al.* Structural and functional characteristics of the Val44Met insulin-like growth factor I missense mutation: correlation with effects on growth and development. *Mol. Endocrinol.* **19**, 711-721 (2005).
10. Bayne, M.L., Applebaum, J., Chicchi, G.G., Miller, R.E. & Cascieri, M.A. The roles of tyrosines 24, 31, and 60 in the high affinity binding of insulin-like growth factor-I to the type 1 insulin-like growth factor receptor. *J. Biol. Chem.* **265**, 15648-15652 (1990).
11. Denley, A., Cosgrove, L.J., Booker, G.W., Wallace, J.C. & Forbes, B.E. Molecular interactions of the IGF system. *Cytokine Growth Factor Rev.* **16**, 421-439 (2005).
12. Collaborative Computing Project, No. 4. The CCP4 suite: programs for protein crystallography. *Acta Crystallogr. D Biol. Crystallogr.* **50**, 760-763 (1994).
